# Dissecting genomic regions and underlying candidate genes in groundnut MAGIC population for drought tolerance

**DOI:** 10.1186/s12870-024-05749-3

**Published:** 2024-11-05

**Authors:** Vinay Sharma, Supriya S. Mahadevaiah, Putta Latha, S. Anjan Gowda, Surendra S. Manohar, Kanchan Jadhav, Prasad Bajaj, Pushpesh Joshi, T. Anitha, Mangesh P. Jadhav, Shailendra Sharma, Pasupuleti Janila, Ramesh S. Bhat, Rajeev K. Varshney, Manish K. Pandey

**Affiliations:** 1 Center of Excellence in Genomics and Systems Biology, International Crops Research Institute for the Semi-Arid Tropics (ICRISAT), Patancheru, Hyderabad, India; 2 Department of Genetics and Plant Breeding, Chaudhary Charan Singh University (CCSU) , Meerut, India; 3 Department of Biotechnology, University of Agricultural Sciences, Dharwad, India; 4 Regional Agricultural Research Station, Acharya N G Ranga Agricultural University (ANGRAU), Tirupati, India; 5 Centre for Crop and Food Innovation, WA State Agricultural Biotechnology Centre, Murdoch University, Murdoch, Australia

**Keywords:** Climate resilient, Drought, Groundnut, Gene, Marker, MAGIC population

## Abstract

**Background:**

Groundnut is mainly grown in the semi-arid tropic (SAT) regions worldwide, where abiotic stress like drought is persistent. However, a major research gap exists regarding exploring the genetic and genomic underpinnings of tolerance to drought. In this study, a multi-parent advanced generation inter-cross (MAGIC) population was developed and evaluated for five seasons at two locations for three consecutive years (2018–19, 2019–20 and 2020–21) under drought stress and normal environments.

**Results:**

Phenotyping data of drought tolerance related traits, combined with the high-quality 10,556 polymorphic SNPs, were used to perform multi-locus model genome-wide association study (GWAS) analysis. We identified 37 significant marker-trait associations (MTAs) (Bonferroni-corrected) accounting, 0.91- 9.82% of the phenotypic variance. Intriguingly, 26 significant MTAs overlap on four chromosomes (Ah03, Ah07, Ah10 and Ah18) (harboring 70% of MTAs), indicating genomic hotspot regions governing drought tolerance traits. Furthermore, important candidate genes associated with leaf senescence (*NAC transcription factor*), flowering (*B3 domain-containing transcription factor*, *Ulp1 protease family*, and *Ankyrin repeat-containing protein*), involved in chlorophyll biosynthesis (*FAR1 DNA-binding domain protein*), stomatal regulation (*Rop guanine nucleotide exchange factor*; *Galacturonosyltransferase*s), and associated with yield traits (*Fasciclin-like arabinogalactan protein 11* and *Fasciclin-like arabinogalactan protein 21*) were found in the vicinity of significant MTAs genomic regions.

**Conclusion:**

The findings of our investigation have the potential to provide a basis for significant MTAs validation, gene discovery and development of functional markers, which could be employed in genomics-assisted breeding to develop climate-resilient groundnut varieties.

**Supplementary Information:**

The online version contains supplementary material available at 10.1186/s12870-024-05749-3.

## Introduction

Groundnut or peanut (*Arachis hypogaea* L.) is an important oilseed, and fodder crop, particularly among small-scale farmers in the SAT regions of Asia and Africa. It is cultivated on 32.72 million hectares worldwide, with an annual production of 53.93 Mt. [1]. In the past few years, global temperature has increased, resulting in drought and high temperature stress in SAT regions [2]. Around 90% of groundnuts is grown under rainfed condition, characterized by irregular rainfall following a severe drought, mostly in Asia and Africa. Studies on climate change prediction in SAT regions have revealed a steady decrease in crop production, posing risks to food availability [3]. Yield loss of up to 33% has been reported due to water stress during the reproductive stage [4, 5]. Notably, groundnut yield has been reported to be reduced by 70% due to water scarcity [6]. In groundnut, flowering and pod filling are the most critical stages under drought stress [7], where prolonged drought can diminish root density and growth, induce leaf curling and shorten the inter-nodal length, consequently impairing water use efficiency and absorption activity, leading to delayed flowering and anthesis, as well as a reduction in pod yield [8]. In addition, drought effects symbiotic nitrogen fixation, which in turn decreases both the yield and quality. Furthermore, it also affects nitrogen content, and digestibility of haulm fodder [9]. Thus, it is important to breed groundnut cultivars that can withstand drought and become ideal for the SAT region, to reduce the adverse effects of drought on groundnut yield and quality. Previous studies have reported that groundnut exhibits substantial variation in drought tolerance-related traits, which is important for crop improvement [10, 11].

Substantial advancements have been made in studying the genetic nature of drought tolerance via comprehensive approaches including physiology and productivity [12, 13]. In groundnut, surrogate traits like transpiration efficiency (TE), specific leaf area (SLA), SPAD chlorophyll meter reading (SCMR), and relative water content (RWC) are considered significant indicators of drought tolerance, influencing yield variability under water stress conditions [14]. RWC and SLA are critical measures of drought tolerance involving water relations and plant response. Maintaining higher RWC and minimizing specific leaf area to mitigate water loss indicated a tolerance to drought. Furthermore, total dry matter content (TDMC) and SCMR are also important traits for genotype selection, exhibiting significant correlations with pod yield under water stress conditions [15]. The conventional breeding approach for improving water stress tolerance in groundnut is restricted by the complex nature of trait, low heritability, and high genotype × environment interaction [16].

In last few years, genomics has made major progress in the exploration of the genetics underpinnings of complex traits [17]. Deployment of genomic resources and advanced breeding strategies, such as genomics-assisted breeding (GAB) [18], genomic selection [17], and rapid generation advancements [19], have improved the efficacy of the groundnut breeding program. Furthermore, the reduced cost of sequencing, availability of reference genomes for cultivated tetraploid groundnut [20–22], genotyping platforms and technologies such as genotyping-by-sequencing [2, 23] and high-density Axiom_*Arachis*' single-nucleotide polymorphism (SNP) arrays [24] have fasten the speed and accuracy of groundnut genomics studies including gene discovery. These advancements have made it possible for high-throughput genotyping to enable development of high-dense genetic maps in groundnut [17]. The constraints of genetic characterization are no longer a bottleneck for discovery of genes for traits of interest. Several QTL mapping studies have been conducted in groundnut for dissecting genetic basis of several traits, using bi-parental populations [25–29], including drought tolerance traits [25, 27], productivity traits [26], heat tolerance related traits [2] and agronomic traits [29]. Drought is a complex trait, consequently, some earlier reports, identify the QTLs for drought traits like transpiration efficiency (TE), SLA, SCMR, relative water content (RWC), shoot dry weight (SDW) [16, 30–32], via low-density genetic maps. Furthermore, there is still need to develop varieties capable to withstand drought while providing substantial yields. This depends on the identification of yield attributing and surrogate traits linked to drought tolerance, as well as the transfer of the genomic regions/genes regulating such traits. Recently, two studies reported a QTL mapping of drought tolerance traits using a dense map [25, 27]. Similarly, efforts have been undertaken to dissect genetic basis of drought tolerance traits in groundnut using high-quality SNPs [6]. QTLs identified by utilizing bi-parental populations have low resolution and usually don’t provide validation in different genetic backgrounds. While LD-based mapping employing natural populations effectively addresses such limitation, but lack of heritability and the contribution of rare alleles are still unresolved. This issue is crucial in crops including groundnut because of its narrow genetic base. Thus, it is critically important to broaden the genetic base by involving multiple parents and introducing maximum genetic diversity that exists in the diverse germplasm. Consequently, there has been a notable rise in the exploitation of diversity panels in genetic dissection in recent decades [33, 34].

In recent studies, the dissection of genetic basis of biotic stress and yield related traits has been employed using specialized mapping populations in groundnut, like multi-parent advanced generation intercross (MAGIC) [35] and nested association mapping (NAM) [36, 37]. Similarly, development of MAGIC populations in *Arabidopsis thaliana* [38] and wheat [39], have provided important insights into the genetic architecture underlying different traits. Multi-parental populations have enhanced mapping resolution and effectiveness. Of multi-parent population, MAGIC is ideally suitable for studying the genomic architecture and provides a robust framework for discovery and characterization of genes for complex traits with higher precision [40]. Additionally, it allows for the integration of higher genetic diversity, and high recombination rates. Trait integration, marker-assisted selection, and precision breeding in groundnuts need an understanding of marker-trait associations.

This study aims to investigate the genetic basis of drought tolerance using the MAGIC population derived from eight groundnut genotypes (ICGV 02022, ICG 7190, ICGV 97183, ICG 3053, ICG 14482, ICG 11515, TAG 24 and ICGV 02266) for developing genomic resources, MTAs and potential genes for improving climate resilience in groundnut.

## Material and methods

### Selection of parents and development of MAGIC population

**Fig. 1:**
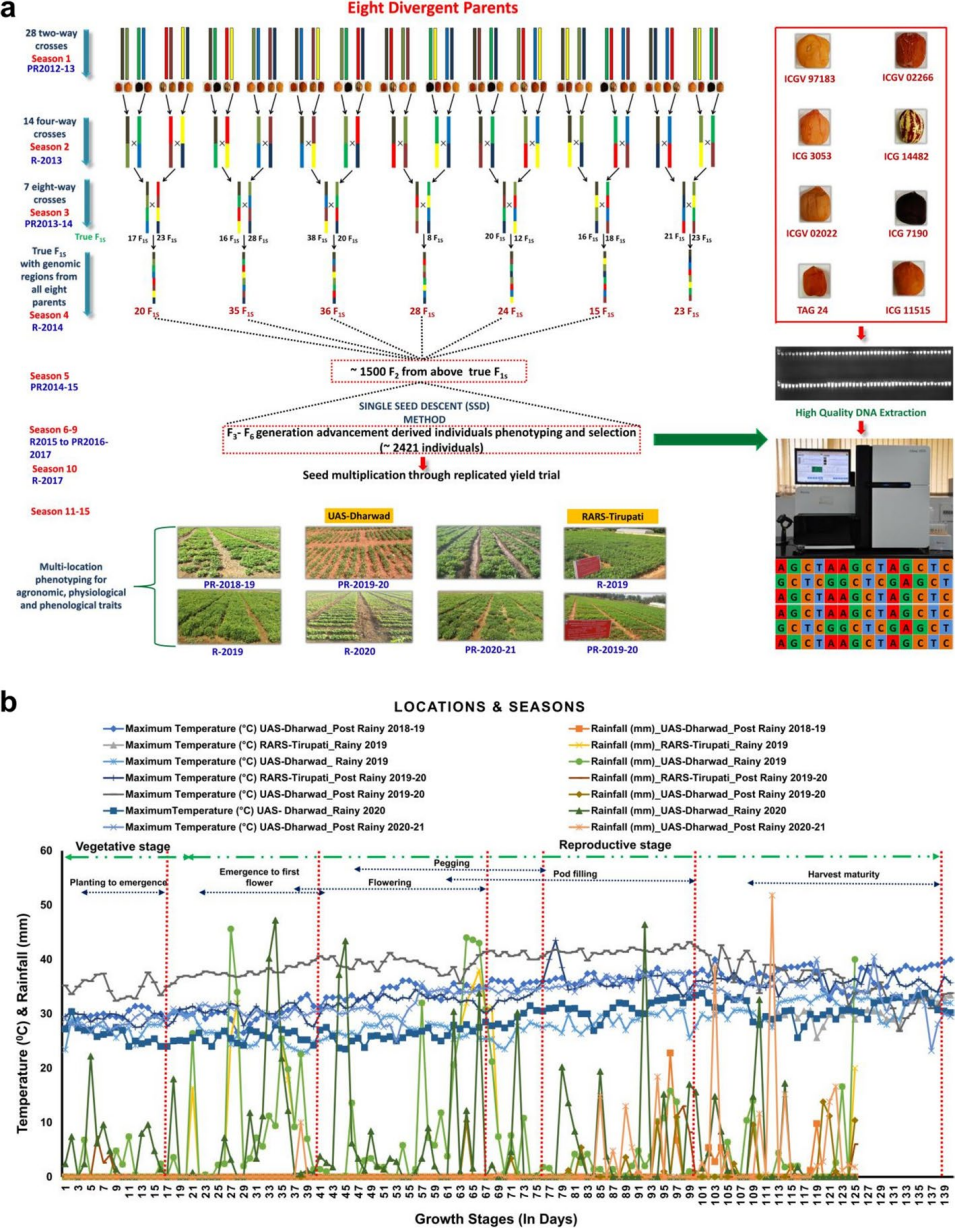
Breeding scheme for the development of groundnut MAGIC population: (**a**) Individual colour represents one of the eight parents; (**b**) Variability in rainfall and temperature across different locations and seasons: The graph depicts the rainfall and daily maximum temperatures recorded during growth stage of groundnut crop over five seasons at two locations (L1: UAS-Dharwad, and L2: RARS-Tirupati)

Eight well-adapted cultivars/elite lines were used as founder parents in the development of MAGIC population. Seeds material were collected from the ICRISAT Genebank, India and detailed information available in Table S1 and at https://genebank.icrisat.org/IND/Referenceset?Crop=Groundnut. These included ICGV 02022 (early maturing with 100–110 days and tolerance to drought), ICGV 97183 (high yielding, early maturing with 100–111 days and drought tolerance) [41]; ICG 7190 (medium duration 110–130 days and moderate canopy temperature) [34]; ICG 3053 (breeding/research material, Origin: India), ICG 14482 (variegated seed colour pattern, traditional cultivar/landrace, Origin: Nigeria), ICG 11515 (medium duration 110–120 days, traditional cultivar/landrace Origin: China)(https://genebank.icrisat.org/IND/Passport?Crop=Groundnut&Location=Passport&mc=Yes); TAG 24 (semi-dwarf variety with high harvest index and better water use efficiency) [42]; and ICGV 02266 (high-yielding, drought tolerant variety with superior haulm quality) [43]. The selected eight parents were inter-crossed in all possible combinations, excluding reciprocals, by generating 28 two-way (Dec 2012–May 2013), 14 four-way (June 2013–Oct 2013) and 7 eight-way crosses (Dec 2013–May 2014) in field (Fig. 1a). All F_1s_ from 7 eight-way crosses were advanced to F_2s_ (June 2014–Oct 2014). A total of > 1500 F_2_ plants were grown during Dec 2014–May 2015 in the field. These were advanced to F_3_ (June -Oct 2015), F_4_ (Dec 2015–May 2016, F_5_ (Jun–Oct 2016), and F_6_ (Dec 2016–May 2017) using the single seed descent (SSD) method in the field. A total of > 2421 F_6_ derived F_7_ MAGIC lines were grown (June–Oct 2017) for seed multiplication at ICRISAT Patancheru. During Post rainy 2018–19, 500 MAGIC RILs were randomly selected from each set of seven crosses for the phenotyping at UAS-Dharwad (Dec 2018-May 2019) (Table S2).

### Multi-environment evaluation and phenotyping for drought tolerance traits

The 500 MAGIC lines (MLs) were evaluated in five seasons for three consecutive years (Table 1 and Fig. 1a) at two field sites, namely, University of Agricultural Sciences (UAS), Dharwad (15.4889′N, 74.9813′E), and Regional Agricultural Research Station (RARS), Acharya N G Ranga Agricultural University (ANGRAU), Tirupati (13.6250′N, 79.3728′E). Three experimental trials were conducted during the post-rainy season (second and last weeks of January) to expose the MLs to water-stress during the pegging and pod formation stage (imposition of mid-season stress), and two trials were conducted during the rainy season (second week of July). From 40 to 80 DAS, irrigation was withheld to impose stress conditions. Prior to 40 DAS, irrigation was provided as needed; and after 80 DAS need-based irrigation continued until harvest. The daily rainfall and temperatures were recorded for growth seasons over a three years (Fig. 1b). A randomized block design was used with a spacing of 22.5 cm × 15 cm at RARS Tirupati, and 30 cm × 10 cm at UAS-Dharwad in two replicates. At each location, standard field procedures were followed over the various evaluation seasons. The Field Scout TDR 350 Soil Moisture Meter was used to measure soil moisture and temperatures.

**Table 1:** Summary of phenotyping data for 18 drought tolerance traits (agronomic, physiological, and phenological traits) in MAGIC population across two locations and five seasons

Traits	PR 2018–19 (S1)	R 2019 (S2)	PR 2019–20 (S3)	R 2020 (S4)	PR 2020–21 (S5)
**Agronomic Traits**					
Total Pod weight (g plant^−1^)	L1	L1	-	-	-
100-seed weight (g)	L1	-	-	L1	L1
Sound mature kernel weight percentage (%)	L1	-	-	L1	L1
Pod yield per plant (g plant^−1^)	-	L2	L2	L1	L1
Total Seed weight (g plant^−1^)	-	-	L2	L1	-
Shelling percentage (%)	-	-	L2	L1	L1
Immature kernel number	-	-	-	L1	L1
Sound mature kernel number	-	-	-	L1	L1
**Physiological and Phenological Traits**					
SPAD chlorophyll meter reading_ Days to 50% flowering	L1	L1	L1	L1	L1
Specific leaf area_ Days to 50% flowering (cm^2^ g^−1^)	-	-	L1	L1	L1
SPAD chlorophyll meter reading (70 DAS)	L1	L1	L1 & L2	L1	L1
Specific leaf area (cm^2^ g^−1^) (70 DAS)	L1	-	L2	L1	-
Plant height (cm)	-	-	-	-	L1
Shoot dry weight (gm)	-	-	-	-	L1
Total dry matter content (gm)	-	-	-	-	L1
Canopy temperature ( 70 DAS) (^0^C)	-	-	L2	-	-
Relative water content (%)	-	-	L2	-	-
Days to 50% flowering`	L1	-	-	-	L1

*PR* post-rainy, *R* rainy, *S* season, *L1* UAS-Dharwad, *L2* RARS-Tirupati, -, not available, *DAS* days after sowing

### Phenotyping for agronomic traits

The MLs were phenotyped for eight agronomic traits such as total pod weight (TPW) (g plant^−1^), 100-seed weight (HSW), sound mature kernel percentage (SMKWP), pod yield per plant (PYPP) (g plant^−1^), total seed weight (TSW) (g plant^−1^), shelling percentage (SP) (%), immature kernel number (IMKN) and sound mature kernel number (SMKN). TPW was measured by weighing the total pods of the plant/plot/m^2^ and expressing the weight in grams. HSW was determined by randomly drawing 100 seeds from each plot yield, weighed and expressed as HSW in grams. For SMKP, 100 kernels were randomly taken from each genotype, and recorded the weight. Bold kernels (smooth seed coat) were separated from 100 kernels and take the weight of the bold seeds. SMKP was measured as the weight of bold kernels divided by the weight of 100 kernels, multiplied by 100 to obtain a percentage. TSW was determined by weighing the total seeds of the plant/plot/m^2^ and expressed as grams. SP was calculated by collecting random sample pods, weighted, shelled, and resulted kernels were also weighted. SP was calculated as the weight of kernels divided by the weight of pods, multiplied by 100 to get percentage. IMKN and SMKN were obtained as the number of mature kernel per plant, number of immature kernel per plant, respectively.

### Phenotyping for phenological and physiological traits

Observations were recorded for phenological and physiological traits like plant height (PH) (cm), days to 50% flowering (DFF), SPAD chlorophyll meter reading (SCMR), specific leaf area (cm^2^ g^−1^) (SLA), shoot dry weight (gm) (SDW), total dry matter content (gm) (TDMC), relative water content (%) (RWC) and canopy temperature (^0^C) (CT) in different growth stages like days to fifty percent flowering (DFF), and 70 days after sowing (DAS). Ten plants were randomly selected, and the average of these plants was used to record data. CT was measured using Infrared Gun (Wahl Heat Spy DHS-10X, Wahl Instruments Inc., USA) and expressed as ^0^C. To determine RWC, leaflets were collected from the third leaf from the top of the primary branch of each genotype, soaked in water for 6 h to gain turgidity. Turgid weights are recorded and dried in hot air oven at 80 °C to a constant weight to record dry weight. RWC was estimated using the formula RWC (%) = [(FW– DW)/(TW– DW)] * 100, where FW stands for fresh weight, DW for dry weight, and TW for turgid weight, as defined by Barrs and Weartherley, [44]. SLA is the ratio of leaf area (cm^2^) to leaf dry weight (g). Leaf area was estimated with a leaf area meter (LICOR model-3100), and leaf samples were oven-dried for at least 48 h at 80^0^C. SCMR (DFF & 70DAS) was determined on all four leaflets of third leaf from the top of main axis using a SPAD meter of Minolta company, NJ, USA (SPAD 502). PH was measured and expressed in cm from the base of the plant to the tip of the terminal bud, with measurements taken from five randomly labelled five plants in each net plot area. For TDMC, plant samples were collected from the field and separated the leaves, stems and root, which were dried in oven at 80 ^0^C for 2 days. The dry weights of oven dried roots, stems, leaves and pods were recorded and expressed as g plant^−1^. Similarly, for SDW, plant samples were collected from the field and separated the leaves, stems and root. These plant parts were dried in oven at 80 ^0^C for 2 days. The dry weights of oven dried stems were recorded and expressed as g plant^−1^. For DFF the number of days taken from sowing to till 50 per cent of plants initiated flowering was counted in three replications of each genotype and expressed as days to 50 percent flowering across all genotypes.

### DNA isolation, library construction, sequencing, and SNP calling

DNA extraction was performed using the Nucleospin Plant II reagent kit (Macherey–Nagel, Düren, Germany; https://guest.link/UM6) from young leaves of each MLs (25–30 days old), as detailed in Pandey et al. [25]. The DNA quality was analysed using a 0.8% agarose gel, and DNA quantification was assessed with Thermo Scientific Nanodrop 2000 Spectrophotometer. Whole-genome re-sequencing (WGRS) of 8 MAGIC parents and 14 other genotypes representing parental lines of mapping populations was performed with the HiSeq 2500 Sequencing System. The library construction, sequencing, and variant analysis was performed using pipeline described by Thudi et al. [45]. The Genotype-by- sequencing (GBS) procedure was executed in accordance with the methodology described by Elshire et al. [46]. DNA was digested using the restriction enzyme *ApeKI* for GBS (recognition G/CWCG site), and T4 DNA ligase ligates barcoded adapters to the DNA fragments. The equal proportion of adapter-ligated fragments utilized in the construction of the library. After removing additional adapters by amplification, these libraries were sequencing on a HiSeq 2500 (Illumina Inc., San Diego, CA, USA).

SNPs were identified using the GBSv2 [47] workflow, which was executed in TASSEL v5 [48], employing the sequence reads from the fastq files. The reference genome used for SNP calling was allotetraploid progenitor (AABB; 2n = 4x = 40) of the cultivated groundnut A. hypogaea var. Shitouqi (http://peanutgr.fafu.edu.cn/index.php) [22]. The in-house script was utilized to verify if the sequencing reads had barcodes exactly matching the expected four-base remnant of the enzyme cut site. The reads were initially checked for barcode information, and reads with 'N' within first 64 bases were rejected. The reads containing barcodes were subsequently demultiplexed based on the barcode sequence. The Burrows-Wheeler Alignment Tool [49] was employed to aligned the remaining good-quality and distinct reads, referred to as tags, against the reference genome. Afterwards, the alignment file underwent the GBSv2 workflow to carry out SNP calling and genotyping. In order to minimize the false positive, samples having less than 80 MB of data were excluded from further analysis. SNPs with low call rates were excluded based on criteria of missing call rate (more than 20%) and minor allele frequency (MAF) less than 5%. Filtering resulted in 10,556 high-quality SNPs across 449 MLs for further analysis.

### Genetic structure and LD decay

The genetic diversity of the MLs was analysed using the high-quality 10,556 polymorphic SNPs. The population structure was evaluated using Bayesian Markov Chain Monte Carlo model (MCMC) in STRUCTURE v2.3.4 [50]. Three independent runs for K values ranging from 1 to 10 were conducted with the parameters using burn-in length of 1,00,000 and 3,00,000 iterations, respectively. Through the log probability of the data [LnP(D)] and delta K (ΔK), which is determined by the rate of change in [LnP(D)] between consecutive K-values, Structure Harvester estimated the K-value. Principal Component Analysis (PCA) was performed with plink (version 1.9) and visualized using the R (https://www.r-project.org). The neighbor-joining (NJ) clustering method in TASSEL 5 [48] was used for construction of tree. The results were visualized using iTOL v6 (https://itol.embl.de). The default parameters were used to calculate LD decay statistics using the software PopLDdecay (v3.41).

### GWAS analysis and identification of candidate genes

For GWAS analysis, this investigation used the phenotyping data generated in five seasons across three consecutive years (excluded the MLs with missing and insufficient data). Finally, 449 MLs comprising 10,556 SNPs were employed for performing GWAS analysis. The multiple loci mixed linear model (MLMM) was implemented using the R/GAPIT 3.0 package to find significant MTAs. The false-positives were corrected using “Bonferroni Correction” (5% level of significance), considering SNPs significant only when they met the correction criteria. Candidate genes associated with the corresponding traits were detected in the vicinity of significant SNP trait associations, examining 1 Mb window (500 kb on either side of SNP) of the genomic regions using annotated reference genome *A. hypogaea* var. Shitouqi (http://peanutgr.fafu.edu.cn/index.php). Moreover, gene expression atlas (AhGEA) [51] was utilized to examine the expression of candidate genes in the tissues.

## Results

### Whole genome resequencing (WGRS) of founder parents and genotyping-by-sequencing of MAGIC lines

A total of 607.89 Gb sequencing data was generated for eight MAGIC founder parents and fourteen additional genotypes, namely ICG14482 (24.24 Gb), ICG7190 (25.37 Gb), ICG11515 (25.72 Gb), ICGV97183 (25.93 Gb), TAG24 (28.41 Gb), ICG3053 (30.31 Gb), ICGV02022 (33.72 Gb), and ICGV02266 (33.74 Gb). 55–437 (18.32 Gb), ICGV 86031 (12.38 Gb), ICGV02251 (28.43 Gb), ICGV12014 (18.04 Gb), ICGV88145 (36.70 Gb), ICGV91278 (34.93 Gb), ICGV97045 (25.77 Gb), ICV89104 (18.59 Gb), GPBD4 (33.53 Gb), U47-5 (18.44 Gb), VRR245 (16.66 Gb), ICG51 (19.32 Gb), GIRNAR-4 (26.94 Gb), GIRNAR-5 (29.62) for the identification of genome-wide structural variations. As a result, a total of 7755.4 million reads of data was generated with a read length ranging from 150 to 250 (Table S3). Across all 22 accessions, 5432.5 million reads were mapped onto the reference genome. Of these, 5,432.5 million mapped reads, 5404.5 million reads were uniquely mapped onto the reference genome, while rest of reads were mapped to the various regions within the genome. The average effective mapping depth was 10.94X, with a range of 4.91X (ICGV 86031) to 18.14X (TAG24). The average genome coverage of each accession against the reference genome was 84.8%, with a range of 46.5% to 97%. Comprehensive data analysis identified a total of 270,232 SNPs. The majority of the SNPs on twenty chromosomes were identified on Ah19 (19,237), while the least were identified on Ah08 (5,998), accounting for 7.11 and 2.21% of the SNPs, respectively (Table S4, Fig. S1). In addition, efforts were undertaken to identify miscellaneous variations, including deletions (DEL), inter-chromosomal variations (CTX), intra-chromosomal variations (ITX) and inversions (INV). Of 5639 structural variations, 1289, 2595, 1634 and 121 were DEL, CTX, ITX and INV respectively (Table S5 and S6). The variants identified through the re-sequencing of 22 accessions offer valuable information into the loci across the groundnut genome, underlining the unique accession signatures and high diversity.

GBS generated 1192.43 million reads (120.68 GB) for 498 MLs and eight parental genotypes. Across the MLs, the number of reads from individual lines varied from 0.54 to 5.90 million (Table S7). On average, 2.35 million reads (0.23 GB) of data were generated for each sample. The sequence reads were mapped to the reference genome *A. hypogaea* var. Shitouqi (http://peanutgr.fafu.edu.cn/index.php), and aligned, cleaned GBS reads were used in the pipeline for SNP calling. A total of 35,347 raw SNPs were detected. Following filtering procedures (samples and SNPs), a total of 10,556 high-quality polymorphic SNPs were used for downstream analysis. These SNPs were distributed across the chromosomes, ranging from 240 (Ah08) to 1721 (Ah18).

### Phenotypic diversity and heritability for drought tolerance traits

**Fig. 2:**
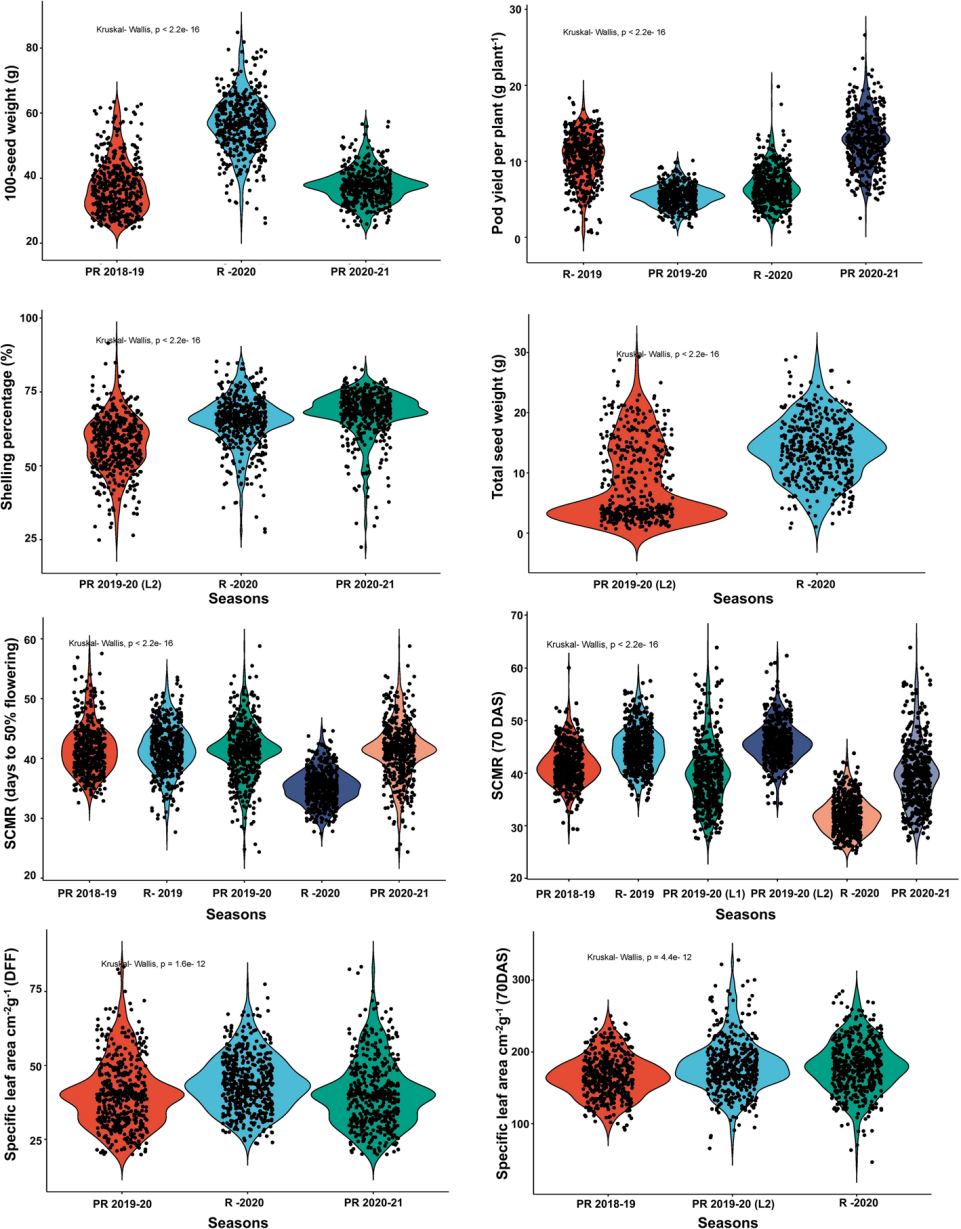
Phenotypic variation in MAGIC population for drought tolerance traits: Violin plot showing variation for agronomic (100-seed weight (g); pod yield per plant (g plant^−1^); total seed weight (g plant^−1^), shelling percentage (%), physiological traits (SPAD chlorophyll meter reading (DFF and 70 DAS); and specific leaf area cm^2^g^−1^ (45 and 70 DAS) for the mean value of the MAGIC population consecutively evaluated 3 years at two locations. Significance is based on Kruskal Wallis test

**Fig. 3:**
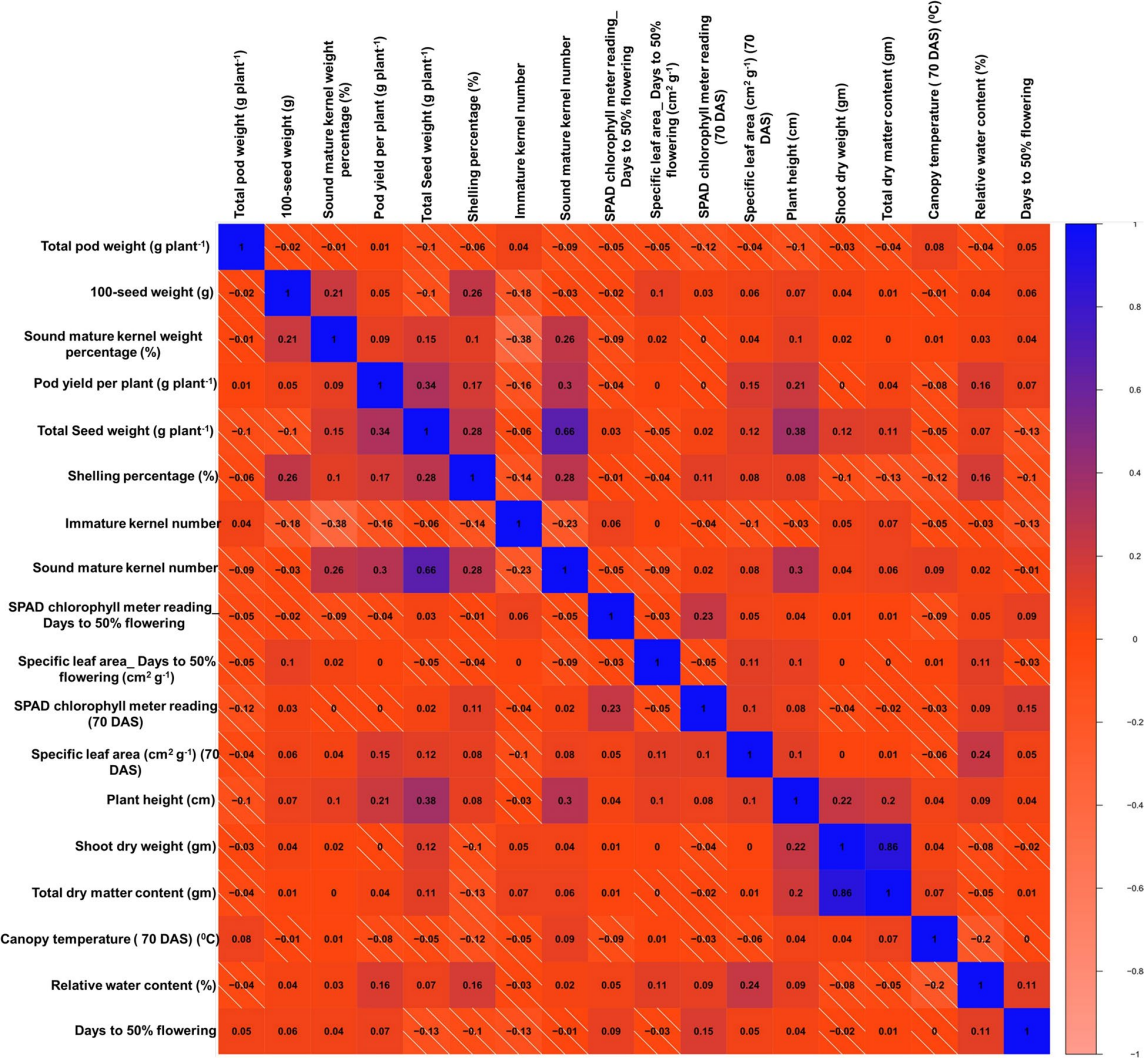
Pearson correlation matrix of drought tolerance traits

The phenotypic observations of the eight agronomic, ten phenological and physiological traits were utilized to determine the possible presence of significant phenotypic variation among MLs. Transgressive segregation was observed for some traits (Fig. 2, Fig. S2). For agronomic traits; HSW of MLs showed significant difference across season (24 g-64.3 g in S1; 20.1 g-85.7 g in S4, and 20.8 g-65.0 g in S5). The average values observed were 37.2 g, 56.5g and 37.6 g, respectively. MLs showed a wide range of variation for TPW (32–272 g plant^-1^ in S1; 21–254 g plant^-1^ in S2), SMKWP (70.0–99.0% in S1; 24.3- 87.0% in S4 and 27.1- 77.2% in S5); PYPP (7.2–21.1 g plant^-1^ in S2; 4.8–11.3 g plant^-1^ in S3; 4.4–24.0 g plant^-1^ in S4 and 3.5–29.0 g plant^-1^ in S5), TSW (2.1–7.3 g plant^-1^ in S3 and 3.2–25.4 g plant^-1^ in in S4), SP (15.7–93.0% in S3; 20.1–94.7% in S4 and 20–84.7% in S5), IMKN (21–56 in S4 and 28–61 in S5), and SMKN (19–42 in S4 and 27–59 in S5). Similarly, the wide genetic diversity created in the MLs produced broad phenotypic variation for phenological and physiological traits; SCMR_DFF (27–73.5 in S1; 21.5–61.2 in S2; 27.3–59.5 in S3; 17.8–57.5 in S4 and 21.5–59.5 in S5), SLA_DFF (17.1–88.3 cm^2^ g^-1^ in S3; 10.9–111.7 cm^2^ g^-1^ in S4 and 16.8–84.3 cm^2^ g^-1^ in S5), SCMR_70DAS (22.8–65.8 in S1; 17.9–44.4 in S2; 25.6–75.0 in S3; 31.6–64.4 in S3 and L2; 19.9–46.9 in S4 and 25.6–75.0 in S5), SLA (39.0–352.9 cm^2^ g^-1^ in S1; 50.9–347.5 cm^2^ g^-1^ in S3 and 28.6–392.0 cm^2^ g^-1^ in S4), PH (14.5–50.5 cm in S5), SDW (0.2–14.5 gm in S5), TDMC (1.1–19.4 gm in S5), CT_70DAS (27–42.00C) and RWC (25.3–87.9%) in S3, and DFF (29–43 in S1 and 31–43 in S5). Traits such as, TPW (g plant^-1^), PYPP (g plant^-1^), TSW (g plant^-1^), IMKN, SLA_DFF (cm^2^ g^-1^), SLA_70 DAS (cm^2^ g^-1^), SDW (gm), TDMC (gm) exhibited higher phenotypic coefficient of variation (PCV) and genotypic coefficient of variation (GCV) (> 20%) across seasons. The broad sense heritability (h^2^) of eight agronomic traits ranged with traits average value 34.5% (IMKN) – 49.5% (TPW (g plant^-1^) and ten phenological and physiological traits ranged from 50% (DFF) – 90% (CT_70 DAS (^0^C) across seasons, indicating that the phenotypes of these traits were primarily determined by genetic factors (Table S8). To estimate the correlation among agronomic, phenological and physiological traits Pearson's correlation analysis was performed. A total of 171 possible correlations were observed, with 17 pairs significantly correlated at the 5% level of probability (Fig. 3). Correlation studies revealed a significant positive correlation between SMKWP and HSW, SMKN and SMKWP, PYPP TSW and SP, suggesting that selection for these attributes would concurrently contribute towards higher yield, and these traits should be given more weightage in the selection process. A negative correlation was observed for TPW and PH, possibly due to the geocarpic nature of groundnut, where aerial flowering and underground pods formation. As the plant height increases, the probability of number of pegs penetrating the soil to make pods decreases. The strong positive correlation was between TDMC and SDW (r = 0.86), while the highest negative correlations were found between IMKN and SMKWP, (r = -0.38). Furthermore, a principal component analysis (PCA) biplot provided an overview of the relationships between different agronomic, phenological and physiological phenotyped under the drought condition. The 45.2% of cumulative variability by PC1 and PC2 was observed (Fig. S3).

### Linkage disequilibrium, genetic diversity and population structure

**Fig. 4:**
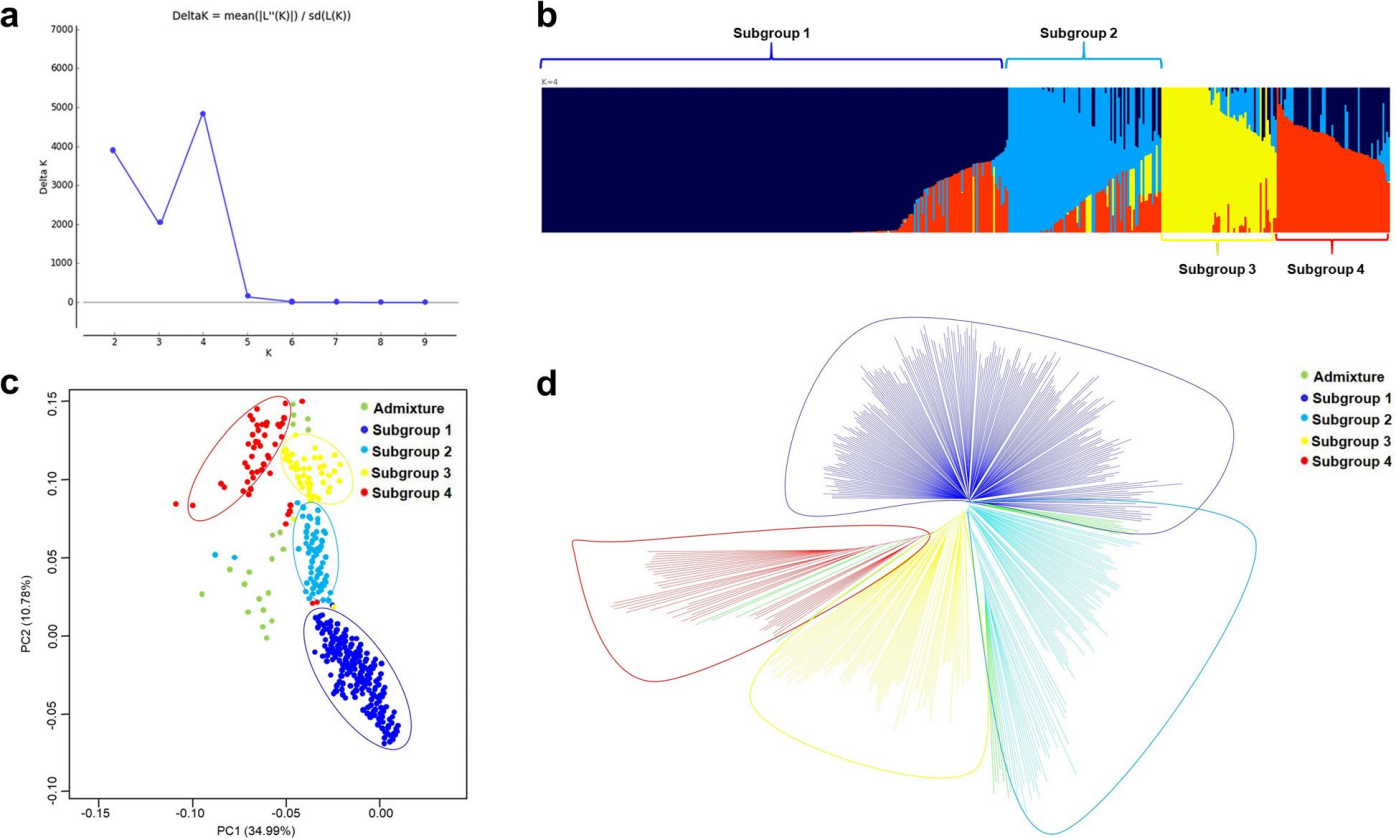
Genetic diversity and population structure of MAGIC population: (**a**) Line graph of delta K over K from 2 to 9. Highest peak was observed at delta K = 4, which indicated the MAGIC population formed four subgroups; (**b**) Population structure using ADMIXTURE analysis (k = 4), optimal with lowest cross-validation error; (**c**) Variation depicted as PCA plot and first two principal components (PC1 and PC2) accounted the maximum overall variability of 45.77%; (**d**) Genetic diversity among MLs using unweighted neighbour-joining tree method, MLs were grouped into four subgroups and admixtures, are denoted in different colour

A total of 10,556 curated SNP markers were retained, each of which had a genotype call rate > 0.80 and MAF 5%. The SNPs were evenly distributed across the 20 chromosomes (Fig. S4a), with an average SNP density of 4.1 SNPs/Mbp. The overall LD decay across the 20 chromosomes was estimated at 180 kbp (Fig. S4b). Population structure among the 449 MLs, derived from seven eight-way crosses, was determined using Admixture, identifying 4 sub-groups (i.e., K = 4) (Fig. 4a and 4b). As shown in Fig. 4b, sub-group I comprised 233 lines, sub-group II contained 88 lines, sub-group III included 71 lines, and sub-group IV consisted of 40 lines, all derived from seven eight-way crosses (Table S9). Allelic admixture among the seven groups derived from eight-way crosses was indicative of substantial proportion of genome reshuffling in the MLs. The PCA also identified the existence of four sub-groups within the 449 MLs. The PCA analysis explained, first two components (PC1 and PC2) accounted for 34.9% and 10.7% of the total genetic variability, respectively (Fig. 4c). Similarly, the NJ tree clustered the 449 MLs into four sub-groups based on the distance matrix (Fig. 4d).

### Genome-wide association (GWAS) analysis

#### GWAS for drought tolerance traits

A total of 37 significant MTAs were detected for 12 traits for five seasons at two locations (L1, and L2). Of 37 MTAs, 21 MTAs were identified for agronomic traits, and 16 MTAs were for phenological and physiological traits (Table 2).

**Table 2:** MTAs detected for drought tolerance traits using multi-locus model in MAGIC population

Trait abbreviations	Season & Location	SNP	Chromosome	Position	P.value	FDR_Adjusted_*P*.values	R^2^
**Agronomic Traits**							
100 seed weight (g)	S1 & L1	S3_44143348	Ah03	44,143,348	4.73E-06	0.002	6.24
	S4 & L1	S3_44143344	Ah03	44,143,344	5.01E-06	0.015	3.14
	S5 & L1	S3_44143348	Ah03	44,143,348	5.34E-06	0.016	2.29
Total pod weight (g plant^−1^)	S1 & L1	S3_44143351	Ah03	44,143,351	4.48E-06	0.001	4.63
	S2 & L1	S3_44143344	Ah03	44,143,344	5.13E-06	0.017	8.92
Pod yield per plant (g plant^−1^)	S3 & L2	S18_9448921	Ah18	9,448,921	4.46E-06	0.001	6.23
	S3 & L2	S20_28650521	Ah20	28,650,521	5.53E-06	0.007	0.92
	S4 & L1	S18_9591418	Ah18	9,591,418	4.78E-06	0.016	3.06
	S5 & L1	S18_9448928	Ah18	9,448,928	7.32E-07	0.007	8.51
	S5 & L1	S18_9591418	Ah18	9,591,418	5.37E-06	0.007	2.08
	S5 & L1	S20_28863197	Ah20	28,863,197	4.81E-06	0.011	5.13
Total seed weight (g plant^−1^)	S5 & L1	S18_9591418	Ah18	9,591,418	4.82E-06	0.016	3.05
	S4 & L1	S18_9601124	Ah18	9,601,124	4.73E-06	0.018	0.97
Shelling percentage (%)	S3 & L2	S19_19362948	Ah19	19,362,948	5.08E-06	0.001	9.14
	S4 & L1	S18_9407788	Ah18	9,407,788	5.26E-06	0.044	0.91
	S5 & L1	S19_19362920	Ah19	19,362,920	5.35E-06	0.016	5.31
Sound mature kernel weight percentage (%)	S4 & L1	S4_93879142	Ah04	93,879,142	7.19E-07	0.006	9.82
	S4 & L1	S18_9755376	Ah18	9,755,376	5.16E-06	0.006	2.08
	S4 & L1	S20_106582234	Ah20	106,582,234	5.44E-06	0.015	5.37
	S5 & L1	S15_39620138	Ah15	39,620,138	5.28E-07	0.001	4.29
	S5 & L1	S18_9781860	Ah18	9,781,860	5.89E-07	0.001	3.31
**Phenological and Physiological traits**							
Days to 50% Flowering (days)	S1 & L1	S16_25939461	Ah16	25,939,461	5.65E-06	0.049	1.73
	S5 & L1	S16_25822849	Ah16	25,822,849	5.54E-06	0.012	2.09
SPAD chlorophyll meter reading _Days to 50% flowering	S3 & L1	S7_14672883	Ah07	14,672,883	6.33E-07	0.006	4.23
	S3 & L1	S7_14694892	Ah07	14,694,892	5.66E-07	0.007	1.89
	S3 & L1	S10_56679980	Ah10	56,679,980	4.85E-06	0.007	4.22
	S3 & L1	S12_52937509	Ah12	52,937,509	4.88E-06	0.007	3.1
	S2 & L1	S7_14694892	Ah07	14,694,892	5.54E-06	0.007	1.21
	S5 & L1	S7_14672883	Ah07	14,672,883	6.33E-07	0.006	6.1
	S5 & L1	S18_9598417	Ah18	9,598,417	5.01E-06	0.031	2.08
SPAD chlorophyll meter reading _70 DAS	S1 & L1	S7_14694292	Ah07	14,694,292	5.14E-06	0.029	2.91
	S3 & L2	S7_14694903	Ah07	14,694,903	4.85E-06	0.001	6.09
	S5 & L1	S10_25185440	Ah10	25,185,440	5.59E-07	0.002	6.26
Specific leaf area _70DAS (cm^2^ g^−1^)	S4 & L1	S10_83718580	Ah10	83,718,580	5.55E-06	0.007	3.24
	S4 & L1	S10_75209380	Ah10	75,209,380	5.13E-06	0.001	5.93
Specific leaf area _Days to 50% flowering (cm^2^ g^−1^)	S5 & L1	S10_75209366	Ah10	75,209,366	5.13E-06	0.014	1.47
Relative water content (%)	S3 & L2	S8_27982767	Ah08	27,982,767	5.24E-06	0.015	3.12

S1: Post-rainy 2018–19; S2: Rainy 2019; S3: Post-rainy 2019–20; S4: Rainy 2020; S5: Post-rainy 2020–21; L1: UAS-Dharwad; L2: RARS-Tirupati; R^2^: Phenotypic variation explained

#### GWAS for agronomic traits

**Fig. 5:**
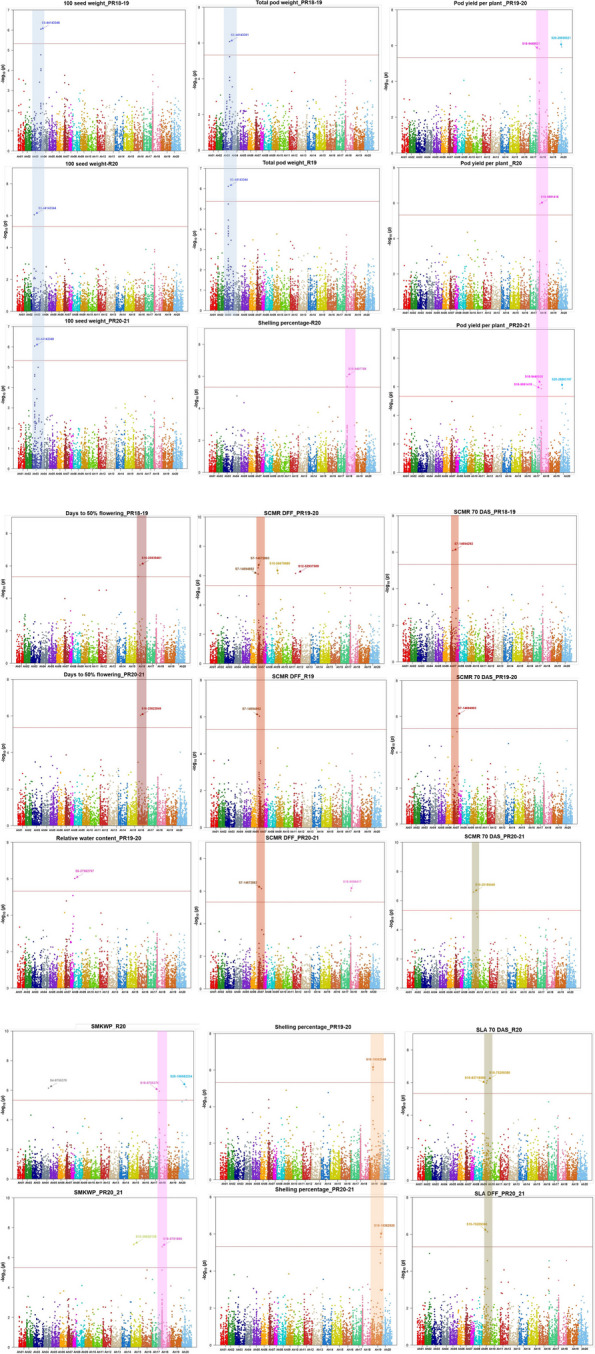
Marker-Trait Associations (MTAs) for drought tolerance traits identified using genome-wide association study in MAGIC population: Manhattan plots showing association in the MAGIC population for agronomic and phenological and physiological traits, Red solid line represents Bonferroni threshold at a significance level of 5%, The x-axis illustrates the SNP position on twenty chromosomes (each represented by different colours) and the y-axis depicts − log (p value) of corresponding SNP in GWAS analysis. SNPs on chromosome highlighted with colours indicate consistently and common significant association for traits across the seasons

Twenty-one MTAs identified for six agronomic traits, explaining 0.91–9.82% PVE with p-value range of 4.73 × 10^–6^ to 7.32 × 10^–7^ (Fig. 5; Fig. S5). Three MTAs detected for HSW on chromosome Ah03 among 3 seasons, explaining the phenotypic variance of 2.29–6.24% during S1, S4 and S5 seasons. For TPW, two MTAs were identified (S3_44143351 and S3_44143344) on same chromosome (Ah03) with PVE of 4.63 and 8.92% over two different seasons (S1 and S2). A total of six MTAs were identified for PYPP, of which four MTAs were identified on chromosome Ah18 and two MTAs (S20_28650521 and S20_28863197) identified on chromosome Ah20. Additionally, the PVE for these six MTAs, accounting 0.92% to 8.51%. Five MTAs were detected on chromosome Ah18 and Ah19 for TSW and SP, accounting 0.91–9.14% PVE during S3, S4 and S5 seasons, respectively. Under two seasons (S4 and S5), five MTAs for SMKWP were detected on chromosome Ah04, Ah15, Ah18 and Ah20, with PVE ranging from 2.08–9.82%. The associated MTAs of agronomic traits were primarily found on chromosomes Ah18 and Ah03, with a fewer detected on Ah20, Ah19, Ah15, Ah04. The highest numbers of MTAs identified for PYPP and SMKWP in S4 and S5 seasons, respectively.

#### GWAS for phenological and physiological traits

For the one phenological and five physiological traits, a total of 16 MTAs were identified (Fig. 5; Fig. S5), which included two MTAs for DFF, seven for SCMR_DFF, three for the SCMR_70DAS, two for SLA_70DAS, one for SLA_DFF, and one for RWC. On chromosome Ah16, two MTAs (S16_25822849 and S16_25939461) for DFF were identified, explaining 1.73–6.26% PVE during S1, S4 and S5 seasons. Ten MTAs were detected for two traits SCMR_DFF and SCMR_70DAS on chromosome Ah07, Ah10, Ah12 and Ah18, with PVE ranging from 1.21–6.26%. Under two seasons (S4 and S5), three MTAs (S10_83718580, S10_75209380, and S10_75209366) for two traits (SLA_70DAS and SLA_DFF) were detected on chromosomes Ah10, with PVE ranging from 1.47% to 5.93%. One MTA (S8_27982767) was detected for RWC on chromosome Ah08, accounting 3.12% PVE.

**Fig. 6:**
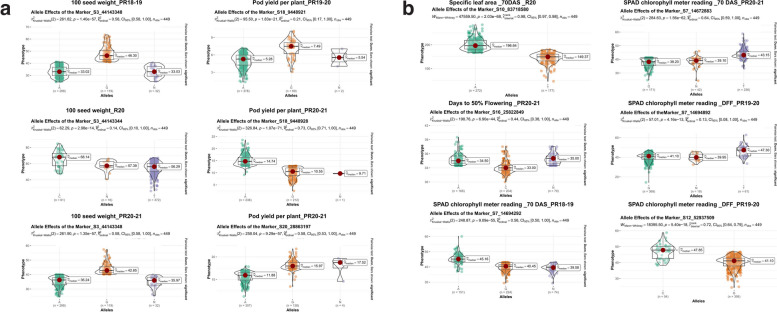
Allelic segregation of associated MTAs detected for drought tolerance traits: Phenotypic variations of allelic differences of associated SNPs for the traits

To visualize the allele effects of a SNP on a trait, we extracted the genotyping calls of significant SNP and phenotyping data for each trait. Phenotypic variations of alleles of associated SNPs were statistically significant and depicted in Fig. 6 and Fig. S6.

#### Candidate genes underlying significant MTAs

**Fig. 7:**
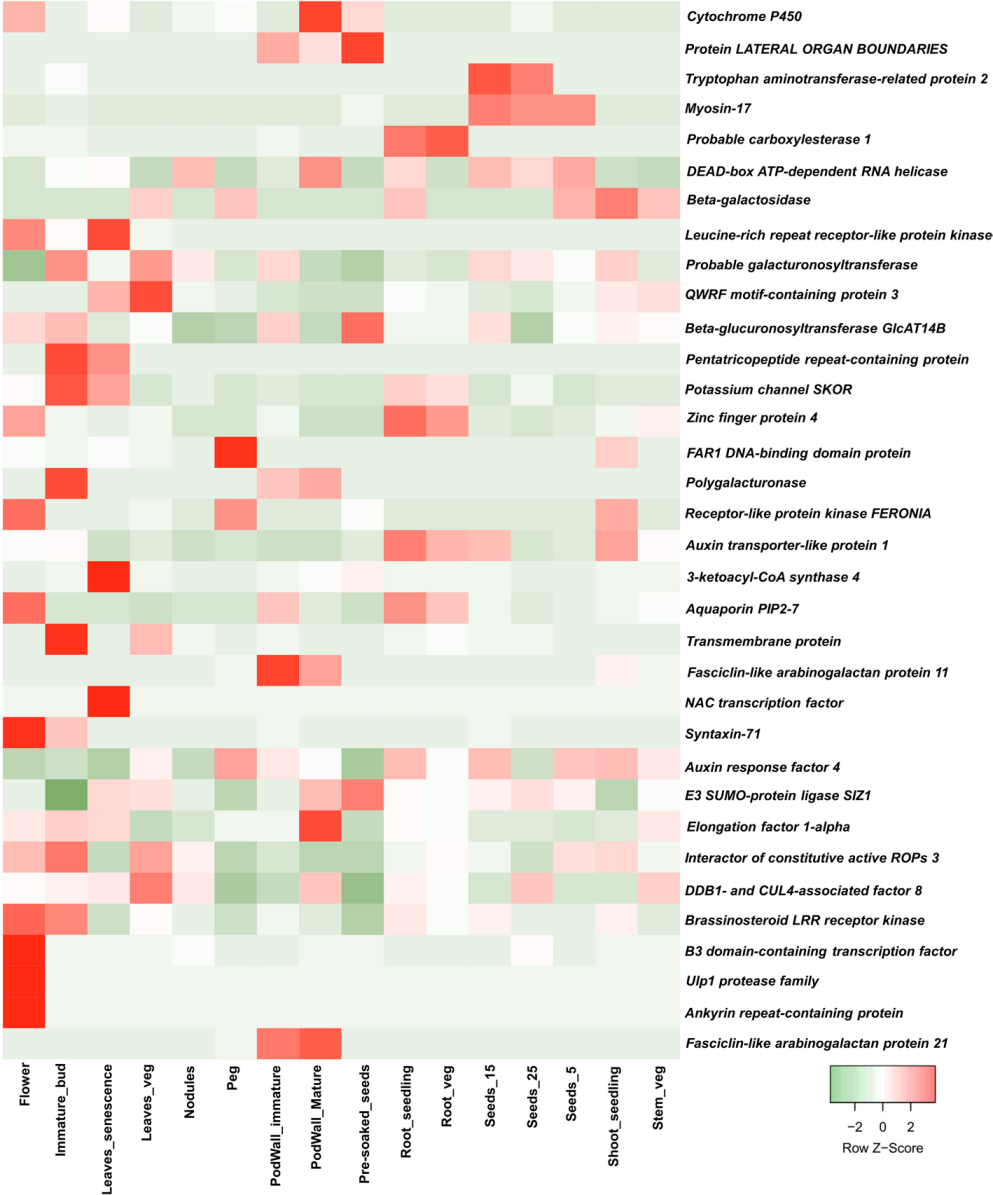
Tissue-specific expression of candidate genes underlying significant MTAs genomic regions: Heatmap shows the expression of 34 candidate genes across 16 different tissues, including flower, immature bud, leaves senescence, leaves veg, nodules, peg, pod wall immature, pod wall mature, pre-soaked seeds, root seedling, root veg, Seeds_15, Seeds_25, Seeds_5, shoot seedling, and stem veg

We investigated a 500 kb region upstream and downstream of the significant MTAs in the annotated reference genome *Arachis hypogaea* var. Shitouqi (http://peanutgr.fafu.edu.cn/index.php) to identify candidate genes associated with drought tolerance traits.; a total of 480 genes were found in 34 MTAs genomic region. Of 480 candidate genes identified, 86 were uncharacterised while an additional 83 with unknown function (Table S10). Functional annotation revealed genes found in the genomic region are linked with biological processes. Thirty-five candidate genes were prioritized according to their functions in controlling drought tolerance traits (Table 3). Moreover, gene expression atlas (AhGEA) was utilized to examine the expression of candidate genes in the tissues. Among the 35 candidate genes identified in this study, 26 genes exhibited differentially expression in at least one tissue during key developmental stages, across 20 tissues (Fig. 7).

**Table 3:** Candidate gene for significant MTAs identified for drought tolerance traits in the MAGIC population

Trait	SNP ID	Chromosome	Gene ID	Position (Start–End) (bp)	Functional Annotation
**HSW, TPW**	S3_44143344; S3_44143348; S3_44143351	Ah03	AH03G23100	43,671,670..43,674,051	*Cytochrome P450*
			AH03G23450	44,082,648..44,085,344	*Protein LATERAL ORGAN BOUNDARIES*
			AH03G23460	44,098,126..44,109,126	*Tryptophan aminotransferase-related protein 2*
			AH03G23640	44,472,040..44,487,712	*Myosin-17*
**SMKWP**	S4_93879142	Ah04	AH04G19090	93,683,461..93,684,312	*Beta-galactosidase*
			AH04G19110	93,912,058..93,915,888	*Leucine-rich repeat receptor-like protein kinase*
**SCMR_DFF, SLA_70DAS**	S7_14672883, S7_14694892, S7_14694292, S7_14694903	Ah07	AH07G10620	14,198,065..14,199,936	*Pentatricopeptide repeat-containing protein*
			AH07G10680	14,337,461..14,344,617	*Potassium channel SKOR*
			AH07G10860	14,635,741..14,637,912	*Zinc finger protein 4*
			AH07G10980	14,829,455..14,830,060	*FAR1 DNA-binding domain protein*
			AH07G11020	14,911,068..14,914,316	*Polygalacturonase*
**RWC**	S8_27982767	Ah08	AH08G13340	27,500,259..27,502,710	*3-ketoacyl-CoA synthase 4*
			AH08G13510	27,847,417..27,849,771	*Aquaporin PIP2-7*
			AH08G13550	27,874,616..27,875,895	*Transmembrane protein*
			AH08G13630	27,942,994..27,944,077	*Fasciclin-like arabinogalactan protein 11*
			AH08G13680	27,977,685..27,979,944	*NAC transcription factor ONAC010*
			AH08G13690	27,982,127..27,983,025	*Syntaxin-71*
			AH08G13730	28,019,641..28,023,425	*Auxin response factor 4*
			AH08G13740	28,024,078..28,032,472	*E3 SUMO-protein ligase SIZ1*
			AH08G13770	28,068,814..28,071,247	*Elongation factor 1-alpha*
			AH08G13830	28,196,453..28,198,830	*Interactor of constitutive active ROPs 3*
			AH08G13890	28,280,194..28,282,987	*DDB1- and CUL4-associated factor 8*
**SLA_70DAS**	S10_83718580	Ah10	AH10G18010	83,666,969..83,670,578	*Brassinosteroid LRR receptor kinase*
**SCMR_DFF**	S10_56679980	Ah10	AH10G15190	56,393,742..56,394,466	*Receptor-like protein kinase FERONIA*
**SCMR_70DAS**	S10_25185440	Ah10	AH10G12170	24,733,907..24,736,057	*Rop guanine nucleotide exchange factor 2*
			AH10G12310	25,423,719..25,429,494	*Auxin transporter-like protein 1*
**DFF**	S16_25822849, S16_25939461	Ah16	AH16G14600	25,497,953..25,500,823	*B3 domain-containing transcription factor*
			AH16G14690	25,576,160..25,578,683	*Ulp1 protease family*
			AH16G14810	25,819,838..25,823,949	*Ankyrin repeat-containing protein*
**PYPP,TSW,SMKWP, SP**	S18_9448921, S18_9591418, S18_9448928, S18_9601124, S18_9407788, S18_9755376, S18_9781860	Ah18	AH18G08770	10,233,052..10,236,486	*Probable galacturonosyltransferase*
			AH18G08780	10,237,345..10,239,790	*QWRF motif-containing protein 3*
			AH18G08800	10,248,622..10,252,201	*Beta-glucuronosyltransferase GlcAT14B*
			AH18G08420	9,817,549..9,818,538	*Fasciclin-like arabinogalactan protein 21*
**PYPP**	S20_28650521, S20_28863197	Ah20	AH20G15910	28,607,531..28,608,601	*Probable carboxylesterase 1*
			AH20G16040	29,244,237..29,244,831	*DEAD-box ATP-dependent RNA helicase*

*TPW* total pod weight, *HSW* 100-seed weight, *SMKWP* sound mature kernel percentage, *PYPP* pod yield per plant, *TSW* total seed weight, *SP* shelling percentage, *DFF* days to 50% flowering, *SCMR_DFF* SPAD chlorophyll meter reading_ days to 50% flowering; SCMR_70DAS: SPAD chlorophyll meter reading_70 days after sowing; SLA_70DAS: specific leaf area_70 days after sowing; RWC: relative water content

## Discussion

Key goal in crop breeding is to find the genes (or sequence variants, including non-coding regions) which are liable for phenotypic variation linked to traits. The comprehensive dissection of the genetic basis of complex traits is primarily influenced by a low diversity of the mapping population, a minor effect of QTL, and a low frequency of the causative variants. Nowadays, it’s possible to creating and harnessing the genetic diversity by developing inbred lines derived from multi-parent cross designs. By introducing several founders with broader genetic and phenotypic diversity, and performing several cycles of inter-crossing and selfing, the number of accumulated recombinant events is increased, resulting in improved mapping accuracy [40]. MAGIC populations have enabled understanding of the genetic architecture underlying many traits. Over the past decade, several research groups have developed MAGIC populations in various crop species like rice [52], cotton [53], tobacco [54], eggplant [55]. One of the most severe stresses which threaten sustainable crop production in SAT regions is water scarcity, as the yields are reduced annually as a result of drought [56]. Integrating drought surrogate traits is important for successful drought tolerance breeding, including groundnut. Previous studies have demonstrated quantitative nature of drought tolerance traits and difficulties in selection for it poses challenges, resulting in conventional breeding approach for developing drought-tolerant crops being laborious and time-consuming [32]. However, progress in breeding and releasing drought-tolerant varieties has been hindered by the complex nature of gene action and combined influence of genotype- environment interactions [31]. Improving the resilience of groundnut cultivars to drought is crucial for improving productivity, as water-stress tolerance is a major constraint. It is crucial to understand that relying solely on phenotypic data for selection is not enough and unreliable due to the substantial influence of environmental variables on the trait. Moreover, understanding the genetic underpinning of physiological, and yield-related traits in groundnut might offers possibilities to develop drought-resilient varieties [4]. WGRS analysis reveals a total of 270,232 SNPs and 5639 other miscellaneous genome wide variations among 22 groundnut genotypes studied. There is a significant amount of variation in the genomes of the founder parents and other parental lines. The primary goal of developing MAGIC populations is to exploit and utilize genetic variations to facilitate crop improvement. As a result, the MLs derived from multiple-parental populations will exhibit significant variation, which can be exploited for gene discovery and allele mining. Our study on a MAGIC population demonstrated wide variability in agronomic, phenological and physiological traits. It clearly indicates that repeated recombination events among the parents resulted in a diversity of several combinations of alleles for the trait, under the existence of distinct genetic backgrounds. The current investigation revealed significant genetic variability in the evaluated agronomic, phenological and physiological traits. Previous studies were also reported similar results for heritability (h^2^) across various locations for physiological, and yield related traits in groundnut [16, 57]. These findings support the understanding of the heritability of complex traits exhibit higher degree of variability in response to varied environmental conditions compared to simple inherited traits. Consequently, the breeding lines selected for specific season/location may not exhibit similar performance in another season/location. The situation poses a substantial challenge for breeders to develop resilient varieties with consistent performance.

The results of correlation indicated significant positive associations among several traits i.e., SMKWP and HSW, SMKN and SMKWP, PYPP, TSW and SP. Furthermore, PYPP and TSW revealed significant positive correlations with the physiological trait PH. This suggests increase in plant height resulted in higher pod and seed weight. As expected, a positive correlation was found between SP and HSW. Highest negative correlation was found between IMKN and SMKWP, with a r value of -0.38 compared to other traits, highlighting the highly influential nature of this trait. These findings correspond with the results reported by Gomes and Almeida Lopes, [58] and Gandhadmath et al. [59]. In addition, a bi-plot resulting from principal component analysis (PCA) depicted the relationship between agronomic, phenological and physiological traits. Significant variation in the performance of MLs across different locations/seasons was observed, attributed to complex nature of traits, and large genotype-environment (G × E) interaction.

In association mapping studies, understanding of the pattern of LD is crucial as it determines the resolution and magnitude of the association analysis within a given population. Our investigation revealed an overall LD decay of 180 kbp. This is most likely due to the occurrence of effective recombination events during the development of the MAGIC population. The genome-wide LD decay varied between 14 and 38 Mbp was found in barley MAGIC [60], while common bean MAGIC showed rapid LD decay (51 to 154 kb) was reported by Diaz et al. [61]. In another investigation, faster LD decay was observed in an African core groundnut collection, possibly due to a slightly less markers [62]. Generally, a large number of recombination’s allowed during the MAGIC development, will result in a decrease in the LD decay, enhancing the population's utility for fine mapping of traits. Examining the genetic structure of a population can provide insights into its origin, composition, and evolutionary history, while also minimizing the false positives among associated markers [63]. Population structure among the 449 MAGIC lines was examined using Admixture, revealing the presence of 4 subgroups (i.e., K = 4). The allelic admixture resulting from eight-way crosses in the MAGIC population showed a large proportion of genome reshuffling. Similarly, population structure was assessed performing PCA and by constructing a neighbour-joining tree, confirming the presence of four subgroups. In this investigation, GWAS analysis detected 37 significant MTAs associated with 12 traits, including 21 for six agronomic traits and 16 for six phenological and physiological traits. Direct selection for yield under drought conditions is efficient; however, it is resource-intensive and lacks stability across varying environments. To address these challenges, physiological and surrogate traits like RWC%, SLA, CT, and SCMR can be used as selection criteria alongside yield related traits [64]. MTAs for HSW and TPW were closely linked and identified on Ah03 between 44,143,344 and 44,143,351 chromosomal regions. Two stable MTAs were identified for HSW on the Ah03 in two different seasons (S1 and S5). This suggested expression of these MTAs was not influenced by environmental factors, as reported in other studies. The MTAs identified for HSW and TPW on chromosomes Ah03 in this investigation were consistent with the previously reported genomic region [25]. Furthermore, MTAs for highly correlated traits, such as; PYPP, TSW, SP, and SMKWP, shared a common chromosome Ah18. In agronomic terms, yield is a key criterion of drought tolerance [65]. Identifying drought-tolerance groundnut genotypes usually rely on biomass production and yield related traits under water stress [66, 67]. Drought-stressed genotypes losses moisture, resulting in impaired physiological activity, adversely impacts overall production and nutritional quality [68]. Total 10 MTAs were pinpointed within 374 kb genomic regions. Similarly, many studies also explored QTL clustering across various traits [2, 69, 70], emphasizing the significance of simultaneous assessment of multiple traits and identifying associated genomic regions to traits of interest. In recent studies, four QTL clusters associated with saturated fatty acid content and three hotspot genomic regions for heat traits were reported in groundnut [2, 70]. Similarly, MAGIC population developed in common bean reported a major QTL-hotspot genomic region on chromosome Pv01 for yield and phenological traits [71]. Two MTAs (S19_19362948 and S19_19362920) for SP with 9.14 and 5.13% PVE were detected on Ah19. In previous studies, a QTL associated with SP was detected on chromosomes A09, B02, Ah10, and Ah20 [2, 72]. For PYPP, two more associations (S20_28650521 and S20_28863197) and one for SMKWP (S20_106582234) were identified on chromosome Ah20. For DFF we identified two MTAs on chromosome Ah16 across two season (S1 and S5) explaining, 1.73% and 2.09% phenotypic variation. Six significant associations for two physiological traits (SCMR_DFF & SCMR_70DAS) (S7_14672883, S7_14694892, S7_14694892, S7_14672883, S7_14694292, and S7_14694903) were co-localized in 22 kb genomic region of Ah07 chromosome. Further, five MTAs were for different physiological traits were identified on Ah10, for instance, SCMR-DFF, SCMR_70DAS, SLA_70DAS, and SLA_DFF. Clustering of MTAs for traits may occur due to changes in gene frequency at closely linked loci, as well as the pleiotropic effects of genes. Moreover, the occurrence of this event may have been influenced by pleiotropy and/or linked genes [73]. One significant MTA (S8_27982767) was detected for RWC% on chromosome Ah08, accounting 3.12% phenotypic variance. Likewise, previous studies have reported QTL for RWC% on same chromosome by QTL mapping [2]. Recent investigation demonstrates traits like SCMR, SLA, and RWC are effective traits for drought tolerance [64, 74]. Several studies reported that, under drought stress, chlorophyll levels in drought-tolerant cultivars were significantly higher compared to those in drought-sensitive genotypes [75]. Consequently, SCMR could be used as reliable basis for assessing drought tolerance, as shown in prior studies [76]. RWC and SLA used as surrogate traits for WUE, which can be effectively measured to detect drought-tolerant genotypes and assess leaf water status [65]. SLA is often used for selecting groundnut genotypes with higher WUE. Genotypes with thicker leaves showed higher WUE, resulting in the inference that SLA acts as reliable surrogate traits [77]. The decrease in RWC during the stress phase is a result of a decrease in water uptake under insufficient soil moisture conditions. RWC, indicates the plant water status. It is a substantial factor of the survival of tissue/organs and metabolic activity [78]. However, selecting drought-tolerant genotypes based on traits such as, SCMR, SLA, and RWC would provide more tolerant lines [78]. Interestingly, 26 significant MTAs (approx. 70% MTAs) were found to overlap for drought tolerance traits on chromosomes Ah03, Ah07, Ah10 and Ah18. These genomic regions seem to be hotspot regions that may contribute to the simultaneous improvement of two or more traits in groundnut breeding. This study provided a comprehensive understanding of the relationships between agronomic, physiological, and phenological traits under water stress. The detected MTAs may facilitate understanding of the genetic underpinnings of genomic regions associated with key traits, hence facilitating the development of drought-tolerant and high-yielding groundnut varieties. Additionally, the markers can be validated and utilized in groundnut breeding programs to facilitate trait integration and gene pyramiding.

We mined candidate genes by the functional annotation of gene models in the vicinity of identified significant MTAs genomic regions potentially associated with drought tolerance traits. Within co-localized genomic region on chromosome Ah03 for HSW and TPW, four genes were identified. The *Cytochrome P450 71A1* (*AH03G23100*) gene identified in this study was reported in regulation of grain size and development in rice [79]. For instance, a gene encoding *Lateral organ boundaries domain* (*AH03G23450*) proteins was found to be linked with improved grain width in wheat [80]. Similarly, the *Tryptophan aminotransferase-related protein 2* (*AH03G23460*) genes have demonstrated a role in increasing yield in wheat [81]. Additionally, the *Myosin-17* (*AH03G23640*) gene was found essential for pollen tube elongation in rice, and involved in seed-setting rate [82]. Likewise, plausible candidate genes underlying the MTAs identified on chromosome Ah07 such as; *Putative pentatricopeptide repeat-containing protein* (*AH07G10620*) and *Zinc finger protein 4* (*AH07G10860*) play a role in the development of chloroplasts in soybean and the regulation of chlorophyll content in pepper [83, 84]. *Potassium channel SKOR* (*AH07G10680*) is known to be involved in promoting efficient photosynthesis [85]. *FAR1 DNA-binding domain protein* (*AH07G10980*) encoding gene play a key role in chlorophyll biosynthesis during deetiolation in Arabidopsis [86]. The *AH07G11020* gene encodes a *Polygalacturonase* that regulates cell wall biosynthesis and leaf morphogenesis in response to water-stress [87]. Moreover, four potential genes were found in the Ah10 chromosome genomic region. *Receptor-like protein kinase FERONIA* (*AH10G15190*) is involved in regulating flowering duration in Arabidopsis [88], while a *Rop guanine nucleotide exchange factor* (*AH10G12170*) is involved in developmental stages such as root and stomatal development, as well as pollen tube growth in Arabidopsis [89], Similarly, *Auxin transporter-like protein* (*AH10G12310*) gene plays various physiological functions in water-stress tolerance and hormonal transport [90]. Another, *Brassinosteroid LRR receptor kinase* (*AH10G18010*) gene is involved in photosynthesis and leaf growth/vascularization in Arabidopsis [91]. Furthermore, several genes were identified underlying the Ah18 chromosome genomic region, including *Probable Galacturonosyltransferase* (*AH18G08770*), *QWRF motif-containing protein 3* (*AH18G08780*), and *Beta-glucuronosyltransferase GlcAT14B* (*AH18G08800*). *Galacturonosyltransferases* are known to role in plant growth, and stomatal development [92], and *QWRF motif-containing protein* is involved in plant fertility and floral organ development in Arabidopsis [93]. An, *Beta-glucuronosyltransferase* protein regulates plant growth, development, reproduction, and stress responses in Arabidopsis [94]. Furthermore, *Beta-galactosidase* (*AH04G19090*) gene was involved in early growth and development stages in orange flowers and fruitlets [95].

Interestingly, *fasciclin-like arabinogalactan protein* 11 (*AH18G08420*) gene found in the present investigation was reported to mediate plant development and cell wall synthesis [96] and control pod shell thickness in groundnut [97]. Additionally, three important genes underlying the MTAs (S16_25822849 and S16_25939461) identified for Days to 50% flowering on chromosome Ah16 were pinpointed. The *B3-Domain Transcription Factor* (*AH16G14600*) gene controls the floral transition [98]. The *Ulp1 protease* (*AH16G14690*) family genes encodes *SMALL UBIQUITIN-RELATED MODIFIER* (*SUMO*), role in regulation of flowering time [99]. Similarly, *Ankyrin repeat-containing protein* (*AH16G14810*) modulates the flowering process through its interaction with FT [100]. The *Probable carboxylesterase* (*AH20G15910*) encoding gene is involved in regulating strigolactone and increasing tillers and branches in maize under drought response [101]. In Arabidopsis, *leucine-rich repeat receptor-like kinase* (*AH04G19110*) is involved in regulating abscisic acid signaling and controls seed maturation, dormancy, stomatal closure, and stress response [102]. Furthermore, *DEAD-box ATP-dependent RNA helicase* (*AH20G16040*) gene are involved in regulating water-stress tolerance and stress-related genes in tomato [103]. Moreover, 11 candidate gene were mined in the vicinity of MTA (S8_27982767) identified for RWC. The *Ketoacyl-CoA synthase* (*AH08G13340*) gene regulates the drought stress response and increases leaf epicuticular wax accumulation [104]. The *Aquaporin PIP2-7* (*AH08G13510*) gene is involved growth, morphology, root architecture and relative water content in Arabidopsis [105]. Genes such as *Transmembrane protein, putative* (*AH08G13550*) and *E3 SUMO Ligase SIZ1* (*AH08G13740*) plays major roles in regulating plant growth and drought responses in Arabidopsis [106, 107]. In barley *NAC transcription factor* (*AH08G13680*) gene is involved senescence of flag leaves [108]. The *Fasciclin-like arabinogalactan protein 21* (*AH08G13630*) gene is involved in the composition of primary cell wall matrix in cotton [109]. Moreover, *Auxin response factor 4* (*AH08G13730*) regulates the stomatal function in response to osmotic stress in tomato [110]. In rice, *Elongation factor 1-alpha* (*AH08G13770*) gene involved in drought tolerance and yield enhancement [111]. The *Syntaxin-71* (*AH08G13690*) gene plays a role in plant development and stress response by regulating pH homeostasis in Arabidopsis [112]. In cotton, *Interactor of constitutive active ROPs 3* (*AH08G13830*) gene is involved in drought tolerance via interaction with GhGGB Protein [113]. Similarly, *DDB1- and CUL4-associated factor 8* (*AH08G13890*) gene plays a role in improving drought tolerance in Arabidopsis [114].

Moreover, gene expression atlas (AhGEA) [51] was utilized to examine the expression of candidate genes in the tissues. This analysis revealed twenty-six genes exhibited differentially expression in at least one tissue during key developmental stages. These genes take part in various processes including photosynthesis, seed emergence, plant architecture, grain number, plant genesis, desiccation mechanisms, and the flowering time. Notably, higher expression levels of the *B3 domain-containing transcription factor*, *Ulp1 protease family*, and *Ankyrin repeat-containing protein* were observed in flower tissue. *Fasciclin-like arabinogalactan protein 11* and *Fasciclin-like arabinogalactan protein 21* exhibited higher expression at all stages of the pod wall, while *NAC transcription factor* showed high expression in leaf senescence stage. These results suggest, its important prioritised genes for precise mapping and cloning to understand their genetics/biological connections of traits. Additionally, examining haplotypes in these genes utilizing diverse germplasm sequencing data could potentially facilitate genetic improvements of these trait. The introgression of detected genes to develop drought-tolerant cultivars through genetic engineering/molecular breeding in an efficient way would provide effective and sustainable solutions to the growing challenges of climate change.

## Conclusion

In this study, we identified 37 significant MTAs associated with 12 drought tolerance traits. Notably, 26 significant MTAs were found to overlap on four chromosomes (Ah03, Ah07, Ah10 and Ah18), indicating genomic hotspot regions that govern traits related to drought tolerance. These hotspot genomic regions collectively provide a basis for simultaneous improvement of different traits; however, precise mapping of these genomic regions is required to facilitate their use for candidate gene cloning and MAS. Moreover, these genomic regions harbour important candidate genes associated with various functions, including leaf senescence (*NAC transcription factor*), regulation of flowering (*B3 domain-containing transcription factor*, *Ulp1 protease family*, and *Ankyrin repeat-containing protein*), chlorophyll biosynthesis (*FAR1 DNA-binding domain protein*), stomatal regulation (*Rop guanine nucleotide exchange factor*; *Galacturonosyltransferases*), and yield traits (*Fasciclin-like arabinogalactan protein 11* and *Fasciclin-like arabinogalactan protein 21*). The *AhGEA* expression atlas revealed the expression of twenty-six potential candidate genes during important stages of development, providing key insights into their unique functions. These candidate genes are useful in the discovery of molecular targets, offering information into the biological pathways underpinning the traits of interest, and understanding the molecular basis of complex traits. Prioritizing these genomic regions in subsequent investigations will help elucidate the drought tolerance mechanism and aid in identifying functional markers. This could accelerate the breeding for drought-tolerant lines/varieties through genomics-assisted breeding.

## Supplementary Information

Supplementary Material 1.

Supplementary Material 2.

## Data Availability

The datasets generated and/or analyzed during this study are openly available on the SRA database under Bioproject accession number PRJNA1133227, and PRJNA1143160.

## References

[1] FAOSTAT (2023) “Food and Agriculture Organization of the United Nations Database of Agricultural Production.” FAO Statistical Databases. http://www.fao.org/faostat/. Accessed 14 January 2023

[2] Sharma V, Gangurde SS, Nayak SN, Gowda AS, Sukanth BS, Mahadevaiah SS, et al. Genetic mapping identified three hotspot genomic regions and candidate genes controlling heat tolerance-related traits in groundnut. Front Plant Sci. 2023;14:1182867. 37287715 10.3389/fpls.2023.1182867 PMC10243373

[3] Howden SM, Soussana JF, Tubiello FN, Chhetri N, Dunlop M, Meinke H. Adapting agriculture to climate change. Proc Natl Acad Sci USA. 2007;104:19691–6. 18077402 10.1073/pnas.0701890104 PMC2148359

[4] Pereira JW, Albuquerque MB, Melo Filho PA, Nogueira RJMC, de Lima LM, Santos RC. Assessment of drought tolerance of peanut cultivars based on physiological and yield traits in a semiarid environment. Agric Water Manag. 2016;166:70–6.

[5] Carvalho MJ, Vorasoot N, Puppala N, Muitia A, Jogloy S. Effects of terminal drought on growth, yield and yield components in valencia peanut genotypes. Sabrao J Breed Genet. 2017;49:270–9.

[6] Manjonda RV, Vorasoot N, Puppala N, Muetia AM, Jogloy S. Reproductive efficiency and yield responses of Valencia peanut genotypes under terminal drought conditions. Khon Kaen Agri J. 2018;46:181–92.

[7] Xiong Jie XJ, Li ShuYu LS, Chen LunLin CL, Zou XiaoYun ZX, Song LaiQiang SL, Zou XiaoFen ZX. Effects of drought stress on physiological traits and yield of different drought-tolerant peanut varieties. Acta Agri Jiangxi. 2016;28:1–5.

[8] Yang X, Luo L, Yu W, Mo B, Liu L. Recent advances in the acclimation mechanisms and genetic improvement of peanut for drought tolerance. Agri Sci. 2019;10:1178–93.

[9] Blümmel M, Ratnakumar P, Vadez V. Opportunities for exploiting variations in haulm fodder traits of intermittent drought tolerant lines in a reference collection of groundnut (Arachis hypogaea L). Field Crops Res. 2012;126:200–6.

[10] Azevedo Neto AD, Nogueira RJ, Melo Filho PA, Santos RC. Physiological and biochemical responses of peanut genotypes to water deficit. J Plant Interact. 2010;5:1–10.

[11] Abady S, Shimelis H, Janila P, Yaduru S, Shayanowako AI, Deshmukh D, Chaudhari S, Manohar SS. Assessment of the genetic diversity and population structure of groundnut germplasm collections using phenotypic traits and SNP markers: Implications for drought tolerance breeding. PLoS ONE. 2021;16:e0259883. 34788339 10.1371/journal.pone.0259883 PMC8598071

[12] Nigam SN, Chandra S, Sridevi KR, Bhukta M, Reddy AGS, Rachaputi NR, et al. Efficiency of physiological trait-based and empirical selection approaches for drought tolerance in groundnut. Ann Appl Biol. 2005;146:433–9.

[13] Ratnakumar P, Vadez V, Nigam SN, Krishnamurthy L. Assessment of transpiration efficiency in peanut (Arachis hypogaea L) under drought using a lysimetric system. Plant Biol. 2009;11:124–30. 19778376 10.1111/j.1438-8677.2009.00260.x

[14] Krishnamurthy L, Vadez V, Devi MJ, Serraj R, Nigam SN, Sheshshayee MS, et al. Variation in transpiration efficiency and its related traits in a groundnut (Arachis hypogaea L) mapping population. Field Crops Res. 2007;103:189–97.

[15] Kalariya KA, Singh AL, Chakraborty K, Ajay BC, Zala PV, Nakar CB, et al. SCMR: a more pertinent trait than SLA in peanut genotypes under transient water deficit stress during summer. Proc Natl Acad Sci India Sect B Biol Sci. 2017;87:579–89.

[16] Faye I, Pandey MK, Hamidou F, Rathore A, Ndoye O, Vadez V, et al. Identification of quantitative trait loci for yield and yield related traits in groundnut (Arachis hypogaea L) under different water regimes in Niger and Senegal. Euphytica. 2015;206:631–47. 26594055 10.1007/s10681-015-1472-6 PMC4643859

[17] Pandey MK, Pandey AK, Kumar R, Nwosu CV, Guo B, Wright GC, et al. Translational genomics for achieving higher genetic gains in groundnut. Theor Appl Genet. 2020;133:1679–702. 32328677 10.1007/s00122-020-03592-2 PMC7214508

[18] Varshney RK, Mohan SM, Gaur PM, Gangarao NVPR, Pandey MK, Bohra A, et al. Achievements and prospects of genomics-assisted breeding in three legume crops of the semi-arid tropics. Biotechnol Adv. 2013;31:1120–34. 23313999 10.1016/j.biotechadv.2013.01.001

[19] Parmar S, Deshmukh DB, Kumar R, Manohar SS, Joshi P, Sharma V, et al. Single seed-based high-throughput genotyping and rapid generation advancement for accelerated groundnut genetics and breeding research. Agronomy. 2021;11:1226.

[20] Bertioli DJ, Jenkins J, Clevenger J, Dudchenko O, Gao D, Seijo G, et al. The genome sequence of segmental allotetraploid peanut *Arachis hypogaea*. Nat Genet. 2019;51:877–84. 31043755 10.1038/s41588-019-0405-z

[21] Chen X, Lu Q, Liu H, Zhang J, Hong Y, Lan H, et al. Sequencing of cultivated peanut, *Arachis hypogaea*, yields insights into genome evolution and oil improvement. Mol Plant. 2019;12:920–34. 30902685 10.1016/j.molp.2019.03.005

[22] Zhuang W, Chen H, Yang M, Wang J, Pandey MK, Zhang C, et al. The genome of cultivated peanut provides insight into legume karyotypes, polyploid evolution and crop domestication. Nat Genet. 2019;51:865–76. 31043757 10.1038/s41588-019-0402-2 PMC7188672

[23] Wang J, Yan C, Shi D, Zhao X, Yuan C, Sun Q, et al. The genetic base for peanut height-related traits revealed by a meta-analysis. Plants. 2021;10:1058. 34070508 10.3390/plants10061058 PMC8227209

[24] Pandey MK, Agarwal G, Kale SM, Clevenger J, Nayak SN, Sriswathi M, et al. Development and evaluation of a high density genotyping ‘Axiom_ *Arachis*’ array with 58 K SNPs for accelerating genetics and breeding in groundnut. Sci Rep. 2017;7:40577. 28091575 10.1038/srep40577 PMC5238394

[25] Pandey MK, Gangurde SS, Sharma V, Pattanashetti SK, Naidu GK, Faye I, et al. Improved genetic map identified major QTLs for drought tolerance-and iron deficiency tolerance-related traits in groundnut. Genes. 2020;12:37. 33396649 10.3390/genes12010037 PMC7824586

[26] Jadhav MP, Gangurde SS, Hake AA, Yadawad A, Mahadevaiah SS, Pattanashetti SK, et al. Genotyping-by-sequencing based genetic mapping identified major and consistent genomic regions for productivity and quality traits in peanut. Front Plant Sci. 2021;12:668020. 34630444 10.3389/fpls.2021.668020 PMC8495222

[27] Ghosh S, Mahadevaiah SS, Gowda SA, Gangurde SS, Jadhav MP, Hake AA, et al. Genetic mapping of drought tolerance traits phenotyped under varying drought stress environments in peanut (Arachis hypogaea L). Euphytica. 2022;218:168.

[28] Sowmya M, Nadaf HL, Naidu GK, Chimmad VP, Mirajkar KK, Shirasawa K. Identification of genomic differences and the candidate genes for drought tolerance in peanut. Euphytica. 2023;219:103.

[29] Joshi P, Soni P, Sharma V, Manohar SS, Kumar S, Sharma S, et al. Genome-wide mapping of quantitative trait loci for yield-attributing traits of peanut. Genes. 2024;15:140. 38397130 10.3390/genes15020140 PMC10888419

[30] Varshney RK, Bertioli DJ, Moretzsohn MDC, Vadez V, Krishnamurthy L, Aruna R, et al. The first SSR-based genetic linkage map for cultivated groundnut (Arachis hypogaea L). Theor Appl Genet. 2009;118:729–39. 19048225 10.1007/s00122-008-0933-x

[31] Ravi K, Vadez V, Isobe S, Mir RR, Guo Y, Nigam SN, et al. Identification of several small main-effect QTLs and a large number of epistatic QTLs for drought tolerance related traits in groundnut (Arachis hypogaea L). Theor Appl Genet. 2011;122:1119–32. 21191568 10.1007/s00122-010-1517-0 PMC3057011

[32] Gautami B, Pandey MK, Vadez V, Nigam SN, Ratnakumar P, Krishnamurthy L, et al. Quantitative trait locus analysis and construction of consensus genetic map for drought tolerance traits based on three recombinant inbred line populations in cultivated groundnut (Arachis hypogaea L). Mol Breed. 2012;30:757–72. 22924017 10.1007/s11032-011-9660-0 PMC3410028

[33] Pandey MK, Upadhyaya HD, Rathore A, Vadez V, Sheshshayee MS, Sriswathi M, et al. Genome-wide association studies for 50 agronomic traits in peanut using the ‘reference set’ comprising 300 genotypes from 48 countries of the semi-arid tropics of the world. PLoS ONE. 2014;9:e105228. 25140620 10.1371/journal.pone.0105228 PMC4139351

[34] Shaibu AS, Sneller C, Motagi BN, Chepkoech J, Chepngetich M. Miko ZL ccy. 2020;10:192.

[35] Wankhade AP, Chimote VP, Viswanatha KP, Yadaru S, Deshmukh DB, Gattu S. Genome-wide association mapping for LLS resistance in a MAGIC population of groundnut (Arachis hypogaea L). Theor Appl Genet. 2023;136:43. 36897383 10.1007/s00122-023-04256-7

[36] Gangurde SS, Thompson E, Yaduru S, Wang H, Fountain JC, Chu Y. Linkage-mapping and genome-wide association study identified two peanut late leaf spot resistance loci, PLLSR-1 and PLLSR-2, using a nested association mapping. Phytopathol. 2024;114(6):1346–55. 10.1094/PHYTO-04-23-0143-R 38669464

[37] Gangurde SS, Wang H, Yaduru S, Pandey MK, Fountain JC, Chu Y. Nested-association mapping (NAM)-based genetic dissection uncovers candidate genes for seed and pod weights in peanut (*Arachis hypogaea*). Plant Biotechnol J. 2020;18:1457–71. 31808273 10.1111/pbi.13311 PMC7206994

[38] Kover PX, Valdar W, Trakalo J, Scarcelli N, Ehrenreich IM, Purugganan MD. A multiparent advanced generation inter-cross to fine-map quantitative traits in Arabidopsis thaliana. PLoS Genet. 2009;5:e1000551. 19593375 10.1371/journal.pgen.1000551 PMC2700969

[39] Huang BE, George AW, Forrest KL, Kilian A, Hayden MJ, Morell MK, et al. A multi-parent advanced generation inter-cross population for genetic analysis in wheat. Plant Biotechnol J. 2012;10:826–39. 22594629 10.1111/j.1467-7652.2012.00702.x

[40] Scott MF, Ladejobi O, Amer S, Bentley AR, Biernaskie J, Boden SA. Multi-parent populations in crops: a toolbox integrating genomics and genetic mapping with breeding. Heredity. 2020;125:396–416. 32616877 10.1038/s41437-020-0336-6 PMC7784848

[41] Hamidou F, Ratnakumar P, Halilou O, Mponda O, Kapewa T, Monyo E, et al. Selection of intermittent drought tolerant lines across years and locations in the reference collection of groundnut (Arachis hypogaea L). Field Crops Res. 2012;126:189–99.

[42] Kale DM, Badigannavar AM, Murty GS. Groundnut variety, TAG 24, with potential for wider adaptability. International Arachis Newsletter. 1999;19:12–3.

[43] Janila P. Support the release of resilient groundnut varieties in target regions in collaboration with NARS. 2017. https://repo.mel.cgiar.org/handle/20.500.11766/6644

[44] Barrs HD, Weatherley PE. A re-examination of the relative turgidity technique for estimating water deficits in leaves. Aust J Biol Sci. 1962;15:413–28.

[45] Thudi M, Samineni S, Li W, Boer MP, Roorkiwal M, Yang Z, et al. Whole genome resequencing and phenotyping of MAGIC population for high resolution mapping of drought tolerance in chickpea. The Plant Genome. 2024;17:e20333. 37122200 10.1002/tpg2.20333 PMC12806880

[46] Elshire RJ, Glaubitz JC, Sun Q, Poland JA, Kawamoto K, Buckler ES, Mitchell SE. A robust, simple genotyping-by-sequencing (GBS) approach for high diversity species. PLoS ONE. 2011;6:e19379. 21573248 10.1371/journal.pone.0019379 PMC3087801

[47] Glaubitz JC, Casstevens TM, Lu F, Harriman J, Elshire RJ, Sun Q. Buckler ES TASSEL-GBS: a high capacity genotyping by sequencing analysis pipeline. PLoS ONE. 2014;9:e90346. 24587335 10.1371/journal.pone.0090346 PMC3938676

[48] Bradbury PJ, Zhang Z, Kroon DE, Casstevens TM, Ramdoss Y, Buckler ES. TASSEL: software for association mapping of complex traits in diverse samples. Bioinfo. 2007;23:2633–5. 10.1093/bioinformatics/btm308 17586829

[49] Li H, Durbin R. Fast and accurate short read alignment with Burrows-Wheeler transform. Bioinfo. 2009;25:1754–60. 10.1093/bioinformatics/btp324 PMC2705234 19451168

[50] Alexander DH, Novembre J, Lange K. Fast model-based estimation of ancestry in unrelated individuals. Genome Res. 2009;19:1655–64. 19648217 10.1101/gr.094052.109 PMC2752134

[51] Sinha P, Bajaj P, Pazhamala LT, Nayak SN, Pandey MK, Chitikineni A. *Arachis hypogaea* gene expression atlas for fastigiata subspecies of cultivated groundnut to accelerate functional and translational genomics applications. Plant Biotechnol J. 2020;18:2187–200. 32167667 10.1111/pbi.13374 PMC7589347

[52] Marrano A, Moyers BT. Scanning the rice Global MAGIC population for dynamic genetic control of seed traits under vegetative drought. Plant Phenome J. 2022;5:e20033.

[53] Wang M, Qi Z, Thyssen GN, Naoumkina M, Jenkins JN, McCarty JC. Genomic interrogation of a MAGIC population highlights genetic factors controlling fiber quality traits in cotton. Commun Biol. 2022;5:60. 35039628 10.1038/s42003-022-03022-7 PMC8764025

[54] Liu Y, Yuan G, Si H, Sun Y, Jiang Z, Liu D, et al. Identification of QTLs associated with agronomic traits in tobacco via a biparental population and an eight-way MAGIC population. Front Plant Sci. 2022;13:878267. 35734263 10.3389/fpls.2022.878267 PMC9207565

[55] Mangino G, Arrones A, Plazas M, Pook T, Prohens J, Gramazio P, Vilanova S. Newly developed MAGIC population allows identification of strong associations and candidate genes for anthocyanin pigmentation in eggplant. Front Plant Sci. 2022;13:847789. 35330873 10.3389/fpls.2022.847789 PMC8940277

[56] Johansen C, Nigam SN. Importance of drought stress and its alleviation in legumes. Crop Sci. 1994;24:17–9.

[57] Painawadee M, Jogloy S, Kesmala T, Akkasaeng C, Patanothai A. Heritability and correlation of drought resistance traits and agronomic traits in peanut (Arachis hypogaea L). Asian J Plant Sci. 2009;8:325–34.

[58] Gomes RLF, de Almeida Lopes ÂC. Correlations and path analysis in peanut. Crop Breed Appl Biotechnol. 2005;5:105–10.

[59] Gandhadmath SS, Vidyashree S, Choudhary R, Motagi BN, Hosamani R, Bharati P, et al. Genetic diversity assessment of groundnut (Arachis hypogaea L) for polyphenol content and antioxidant activity: unlocking the nutritional potential. J Plant Biochem Biotechnol. 2024;33:237–47.

[60] Novakazi F, Krusell L, Jensen JD, Orabi J, Jahoor A, Bengtsson T. You had me at “MAGIC”!: four barley MAGIC populations reveal novel resistance QTL for powdery mildew. Genes. 2020;11:1512. 33352820 10.3390/genes11121512 PMC7766815

[61] Diaz S, Ariza-Suarez D, Izquierdo P, Lobaton JD, de La Hoz JF, Acevedo F. Genetic mapping for agronomic traits in a MAGIC population of common bean (Phaseolus vulgaris L) under drought conditions. BMC Genom. 2020;21:1–20. 10.1186/s12864-020-07213-6 PMC7670608 33198642

[62] Achola E, Wasswa P, Fonceka D, Clevenger JP, Bajaj P, Ozias-Akins P. Genome-wide association studies reveal novel loci for resistance to groundnut rosette disease in the African core groundnut collection. Theor Appl Genet. 2023;136:35. 36897398 10.1007/s00122-023-04259-4 PMC10006280

[63] Guo M, Deng L, Gu J, Miao J, Yin J, Li Y, et al. Genome-wide association study and development of molecular markers for yield and quality traits in peanut (Arachis hypogaea L). BMC Plant Biol. 2024;24:244. 38575936 10.1186/s12870-024-04937-5 PMC10996145

[64] Hampannavar MR, Khan H. Association study of morphological and physiological traits with yield in groundnut genotypes under terminal drought condition. IJCMAS. 2019;8:668–78.

[65] Dang P, Patel J, Sorensen R, Lamb M, Chen CY. Genome-wide association analysis identified Quantitative Trait Loci (QTLs) underlying drought-related traits in cultivated peanut (Arachis hypogaea L). Genes. 2024;15:868. 39062647 10.3390/genes15070868 PMC11276114

[66] Duarte EA, Melo Filho PD, Santos RC. Agronomic characteristics and harvest index of different peanut genotypes subjected to water stress. Braz J Agric Environ Enginee. 2013;17:843–7.

[67] Santos RC, Rêgo GM, da Silva AP, Vasconcelos JO, Coutinho JL, Melo Filho PD. Productivity of advanced peanut lines under rainfed conditions in Northeastern Brazil. Braz J Agric Environ Enginee. 2010;14:589–93.

[68] Songsri P, Jogloy S, Vorasoot N, Akkasaeng C, Patanothai A, Holbrook CC. Root distribution of drought-resistant peanut genotypes in response to drought. J Agron Crop Sci. 2008;194:92–103.

[69] Deng X, Gong J, Liu A, Shi Y, Gong W, Ge Q, et al. QTL mapping for fiber quality and yield-related traits across multiple generations in segregating population of CCRI 70. J Cotton Res. 2019;2:1–10.

[70] Liu N, Chen H, Huai D, Xia F, Huang L, Chen W, et al. Four QTL clusters containing major and stable QTLs for saturated fatty acid contents in a dense genetic map of cultivated peanut (Arachis hypogaea L). Mol Breed. 2019;39:1–14.

[71] Diaz S, Ariza-Suarez D, Ramdeen R, Aparicio J, Arunachalam N, Hernandez C, et al. Genetic architecture and genomic prediction of cooking time in common bean (Phaseolus vulgaris L). Front Plant Sci. 2021;11:622213. 33643335 10.3389/fpls.2020.622213 PMC7905357

[72] Luo H, Pandey MK, Khan AW, Guo J, Wu B, Cai Y, et al. Discovery of genomic regions and candidate genes controlling shelling percentage using QTL-seq approach in cultivated peanut (Arachis hypogaea L). Plant Biotechnol J. 2019;17:1248–60. 30549165 10.1111/pbi.13050 PMC6576108

[73] Smith JM, Haigh J. The hitch-hiking effect of a favourable gene. Genetics Res. 1974;23:23–35. 4407212

[74] Banavath JN, Chakradhar T, Pandit V, Konduru S, Guduru KK, Akila CS, Podha S, Puli CO. Stress inducible overexpression of AtHDG11 leads to improved drought and salt stress tolerance in peanut (Arachis hypogaea L). Front Chem. 2018;2:6–34. 10.3389/fchem.2018.00034 PMC5840212 29552555

[75] Zhou R, Yu X, Ottosen CO, Rosenqvist E, Zhao L, Wang Y, et al. Drought stress had a predominant effect over heat stress on three tomato cultivars subjected to combined stress. BMC Plant Biol. 2017;17:1–3. 28122507 10.1186/s12870-017-0974-x PMC5264292

[76] Zaefyzadeh M, Quliyev RA, Babsyeva S, Abbasov MA. The effect of the interaction between genotypes and drought stress on the superoxide dismutase and chlorophyll content in durum wheat landraces. Turkish J Bio. 2009;33:1–7.

[77] Latha P, Anitha T, Kumar AN, Madhuri KN, Vasanthi RP, Reddy DM, et al. Phenotyping RIL population to identify water deficit tolerant lines in groundnut (Arachis hypogaea L). Legume Res. 2022;1:7.

[78] Kalariya KA, Singh AL, Chakraborty K, Patel CB, Zala PV. Relative water content as an index of permanent wilting in groundnut under progressive water deficit stress. Elec J Environ Sci. 2015;8:17.

[79] Usman B, Nawaz G, Zhao N, Liu Y, Li R. Generation of high yielding and fragrant rice (Oryza sativa L) lines by CRISPR/Cas9 targeted mutagenesis of three homoeologs of cytochrome P450 gene family and OsBADH2 and transcriptome and proteome profiling of revealed changes triggered by mutations. Plants. 2020;9:788. 32586052 10.3390/plants9060788 PMC7355857

[80] Wang H, Han X, Fu X, Sun X, Chen H, Wei X, et al. Overexpression of *TaLBD16-4D* alters plant architecture and heading date in transgenic wheat. Front Plant Sci. 2022;13:911993. 36212357 10.3389/fpls.2022.911993 PMC9533090

[81] Shao A, Ma W, Zhao X, Hu M, He X, Teng W, et al. The auxin biosynthetic Tryptophan Aminotransferase Related TaTAR2 1–3A increases grain yield of wheat. Plant Physiol. 2017;174:2274–88. 28626005 10.1104/pp.17.00094 PMC5543937

[82] Hong WJ, Kim EJ, Yoon J, Silva J, Moon S, Min CW, et al. A myosin XI adaptor, TAPE, is essential for pollen tube elongation in rice. Plant Physiol. 2022;190:562–75. 35736513 10.1093/plphys/kiac299 PMC9434255

[83] Feng X, Yang S, Zhang Y, Zhiyuan C, Tang K, Li G, et al. *GmPGL2*, encoding a pentatricopeptide repeat protein, is essential for chloroplast RNA editing and biogenesis in soybean. Front Plant Sci. 2021;12:690973. 34567023 10.3389/fpls.2021.690973 PMC8458969

[84] Borovsky Y, Monsonego N, Mohan V, Shabtai S, Kamara I, Faigenboim A. The zinc-finger transcription factor *CcLOL1* controls chloroplast development and immature pepper fruit color in Capsicum chinense and its function is conserved in tomato. The Plant J. 2019;99:41–55. 30828904 10.1111/tpj.14305

[85] Checchetto V, Segalla A, Allorent G, La Rocca N, Leanza L, Giacometti GM. Thylakoid potassium channel is required for efficient photosynthesis in cyanobacteria. Proc Natl Acad Sci USA. 2012;109:11043–8. 22711813 10.1073/pnas.1205960109 PMC3390830

[86] Tang W, Wang W, Chen D, Ji Q, Jing Y, Wang H, et al. Transposase-derived proteins FHY3/FAR1 interact with Phytochrome-interacting factor1 to regulate chlorophyll biosynthesis by modulating HEMB1 during deetiolation in Arabidopsis. Plant Cell. 2012;24:1984–2000. 22634759 10.1105/tpc.112.097022 PMC3442582

[87] Zhang G, Hou X, Wang L, Xu J, Chen J, Fu X, et al. Photo-sensitive Leaf rolling 1 encodes a polygalacturonase that modifies cell wall structure and drought tolerance in rice. New Phytol. 2021;229:890–901. 32858770 10.1111/nph.16899

[88] Wang L, Yang T, Lin Q, Wang B, Li X, Luan S, et al. Receptor kinase FERONIA regulates flowering time in Arabidopsis. BMC Plant Biol. 2020;20:1–16. 31948398 10.1186/s12870-019-2223-y PMC6966814

[89] Gu Y, Li S, Lord EM, Yang Z. Members of a novel class of Arabidopsis Rho guanine nucleotide exchange factors control Rho GTPase-dependent polar growth. Plant Cell. 2006;18:366–81. 16415208 10.1105/tpc.105.036434 PMC1356545

[90] Remy E, Cabrito TR, Baster P, Batista RA, Teixeira MC, Friml J. A major facilitator superfamily transporter plays a dual role in polar auxin transport and drought stress tolerance in Arabidopsis. Plant Cell. 2013;25:901–26. 23524662 10.1105/tpc.113.110353 PMC3634696

[91] Oh MH, Sun J, Oh DH, Zielinski RE, Clouse SD, Huber SC. Enhancing Arabidopsis leaf growth by engineering the BRASSINOSTEROID INSENSITIVE1 receptor kinase. Plant Physiol. 2011;157:120–31. 21795582 10.1104/pp.111.182741 PMC3165863

[92] Guo H, Xiao C, Liu Q, Li R, Yan Z, Yao X, et al. Two galacturonosyltransferases function in plant growth, stomatal development, and dynamics. Plant Physiol. 2021;187:2820–36. 34890462 10.1093/plphys/kiab432 PMC8644590

[93] Ma H, Xu L, Fu Y, Zhu L. Arabidopsis *QWRF1* and *QWRF2* redundantly modulate cortical microtubule arrangement in floral organ growth and fertility. Front Cell Develop Biol. 2021;9:634218. 10.3389/fcell.2021.634218 PMC7901996 33634133

[94] Zhang Y, Held MA, Showalter AM. Elucidating the roles of three β-glucuronosyltransferases (GLCATs) acting on arabinogalactan-proteins using a CRISPR-Cas9 multiplexing approach in Arabidopsis. BMC Plant Biol. 2020;20:1–20. 32423474 10.1186/s12870-020-02420-5 PMC7236193

[95] Wu Z, Burns JK. A β-galactosidase gene is expressed during mature fruit abscission of ‘Valencia’orange (*Citrus sinensis*). J Exp Bot. 2004;55:1483–90. 15208347 10.1093/jxb/erh163

[96] Ito S, Suzuki Y, Miyamoto K, Ueda J, Yamaguchi I. AtFLA11, a fasciclin-like arabinogalactan-protein, specifically localized in screlenchyma cells. Biosci Biotechnol Biochem. 2005;69:1963–9. 16244449 10.1271/bbb.69.1963

[97] Liu H, Zheng Z, Sun Z, Qi F, Wang J, Wang M, et al. Identification of two major QTLs for pod shell thickness in peanut (Arachis hypogaea L) using BSA-seq analysis. BMC Genom. 2024;25:65. 10.1186/s12864-024-10005-x PMC10790476 38229017

[98] Jing Y, Guo Q, Lin R. The B3-domain transcription factor VAL1 regulates the floral transition by repressing Flowering Locus T. Plant Physiol. 2019;181:236–48. 31289216 10.1104/pp.19.00642 PMC6716252

[99] Murtas G, Reeves PH, Fu YF, Bancroft I, Dean C, Coupland G. A nuclear protease required for flowering-time regulation in Arabidopsis reduces the abundance of SMALL UBIQUITIN-RELATED MODIFIER conjugates. Plant Cell. 2003;15:2308–19. 14507998 10.1105/tpc.015487 PMC197297

[100] Tang Q, Zhao YN, Luo S, Lu S. AKR2A is involved in the flowering process of Arabidopsis thaliana. Plant Signal Behavior. 2022;17:2100685. 10.1080/15592324.2022.2100685 PMC9311315 35867124

[101] Roesler K, Lu C, Thomas J, Xu Q, Vance P, Hou Z, et al. Arabidopsis carboxylesterase 20 binds strigolactone and increases branches and tillers when ectopically expressed in Arabidopsis and maize. Front Plant Sci. 2021;12:639401. 33986761 10.3389/fpls.2021.639401 PMC8110907

[102] Osakabe Y, Maruyama K, Seki M, Satou M, Shinozaki K, Yamaguchi-Shinozaki K. Leucine-rich repeat receptor-like kinase1 is a key membrane-bound regulator of abscisic acid early signaling in Arabidopsis. Plant Cell. 2005;17:1105–19. 15772289 10.1105/tpc.104.027474 PMC1087989

[103] Zhu M, Chen G, Dong T, Wang L, Zhang J, Zhao Z, Hu Z. SlDEAD31, a putative DEAD-box RNA helicase gene, regulates salt and drought tolerance and stress-related genes in tomato. PLoS ONE. 2015;10:e0133849. 26241658 10.1371/journal.pone.0133849 PMC4524616

[104] Lokesh U, Venkatesh B, Kiranmai K, Nareshkumar A, Amarnathareddy V, Rao GL. Overexpression of ß-Ketoacyl Co-A Synthase1 gene improves tolerance of drought susceptible groundnut (Arachis hypogaea L) cultivar K-6 by increased leaf epicuticular wax accumulation. Front Plant Sci. 2019;9:1869. 30687340 10.3389/fpls.2018.01869 PMC6336926

[105] Israel D, Lee SH, Robson TM, Zwiazek JJ. Plasma membrane aquaporins of the PIP1 and PIP2 subfamilies facilitate hydrogen peroxide diffusion into plant roots. BMC Plant Biol. 2022;22:566. 36471241 10.1186/s12870-022-03962-6 PMC9721007

[106] Zhou Y, Wang B, Yuan F. The role of transmembrane proteins in plant growth, development, and stress responses. Inter J Mol Sci. 2022;23:13627. 10.3390/ijms232113627 PMC9655316 36362412

[107] Catala R, Ouyang J, Abreu IA, Hu Y, Seo H, Zhang X, et al. The Arabidopsis E3 SUMO ligase SIZ1 regulates plant growth and drought responses. Plant Cell. 2007;19:2952–66. 17905899 10.1105/tpc.106.049981 PMC2048692

[108] Christiansen MW, Gregersen PL. Members of the barley NAC transcription factor gene family show differential co-regulation with senescence-associated genes during senescence of flag leaves. J Exp Bot. 2014;65:4009–22. 24567495 10.1093/jxb/eru046 PMC4106437

[109] Huang GQ, Gong SY, Xu WL, Li W, Li P, Zhang CJ, et al. A fasciclin-like arabinogalactan protein, GhFLA1, is involved in fiber initiation and elongation of cotton. Plant Physiol. 2013;161:1278–90. 23349362 10.1104/pp.112.203760 PMC3585596

[110] Bouzroud S, Gasparini K, Hu G, Barbosa MAM, Rosa BL, Fahr M, et al. Down regulation and loss of auxin response factor 4 function using CRISPR/Cas9 alters plant growth, stomatal function and improves tomato tolerance to salinity and osmotic stress. Genes. 2020;11:272. 32138192 10.3390/genes11030272 PMC7140898

[111] Gu Q, Kang J, Gao S, Zhao Y, Yi H, Zha X. Eukaryotic translation elongation factor *OsEF1A* positively regulates drought tolerance and yield in rice. Plants. 2023;12:2593. 37514208 10.3390/plants12142593 PMC10383209

[112] Zhang H, Zhou J, Kou X, Liu Y, Zhao X, Qin G, et al. Syntaxin of plants71 plays essential roles in plant development and stress response via regulating pH homeostasis. Front Plant Sci. 2023;14:1198353. 37342145 10.3389/fpls.2023.1198353 PMC10277689

[113] Hu Z, Lei J, Dai P, Liu C, Wugalihan A, Liu X, et al. A small Gtp-binding protein GhROP3 interacts with GhGGB protein and negatively regulates drought tolerance in cotton (Gossypium hirsutum L). Plants. 2022;11:1580. 35736735 10.3390/plants11121580 PMC9227279

[114] Lian Y, Lian C, Wang L, Li Z, Yuan G, Xuan L, et al. SUPPRESSOR OF MAX2 LIKE 6, 7, and 8 interact with DDB1 Binding WD Repeat Domain Hypersensitive to ABA Deficient 1 to regulate the drought tolerance and target Sucrose nonfermenting 1 related protein kinase 23 to abscisic acid response in Arabidopsis. Biomol. 2023;13:1406. 10.3390/biom13091406 PMC10526831 37759806

