# Supplementary material for: Dissecting genomic regions and underlying candidate genes in groundnut MAGIC population for drought tolerance
Source: BMC Plant Biol. 2024 Nov 5;24:1044. doi: 10.1186/s12870-024-05749-3 (PMC11536578; doi:10.1186/s12870-024-05749-3)
Supplement: Supplementary file 1 — Supplementary Material 1. [file 12870_2024_5749_MOESM1_ESM.docx]

**Supplementary Figures**

**
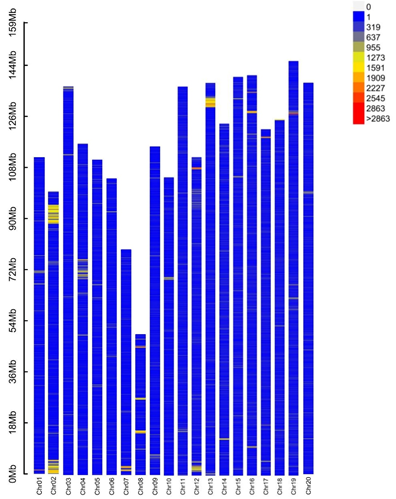
**

**Figure S1. SNP density plot for highly polymorphic 270232 SNPs across twenty chromosomes of 8 founder and 14 parental genotypes**

**Figure S2.** Phenotypic variation in MAGIC population for different drought tolerance-related traits: Violin plot showing variation for agronomic (pod yield per plant (g); sound mature kernel weight percentage; sound mature kernel number, immature kernel number, and phenological (days to 50% flowering) and physiological traits (canopy temperature (70 DAS); plant height (cm); shoot dry weight (gm) and total dry matter content (gm) and relative water content percentage (%). Significance is based on Kruskal Wallis test


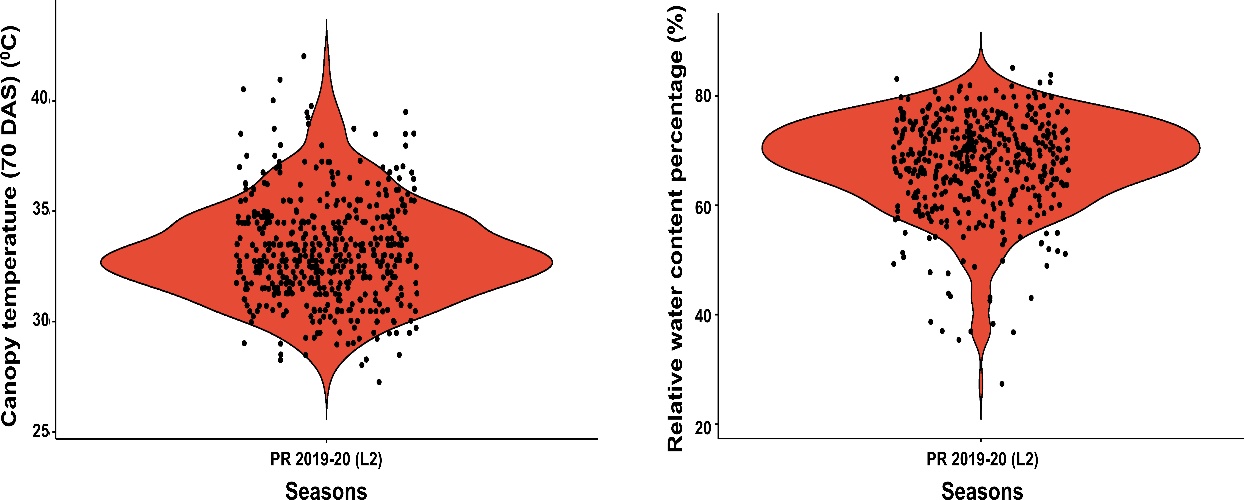


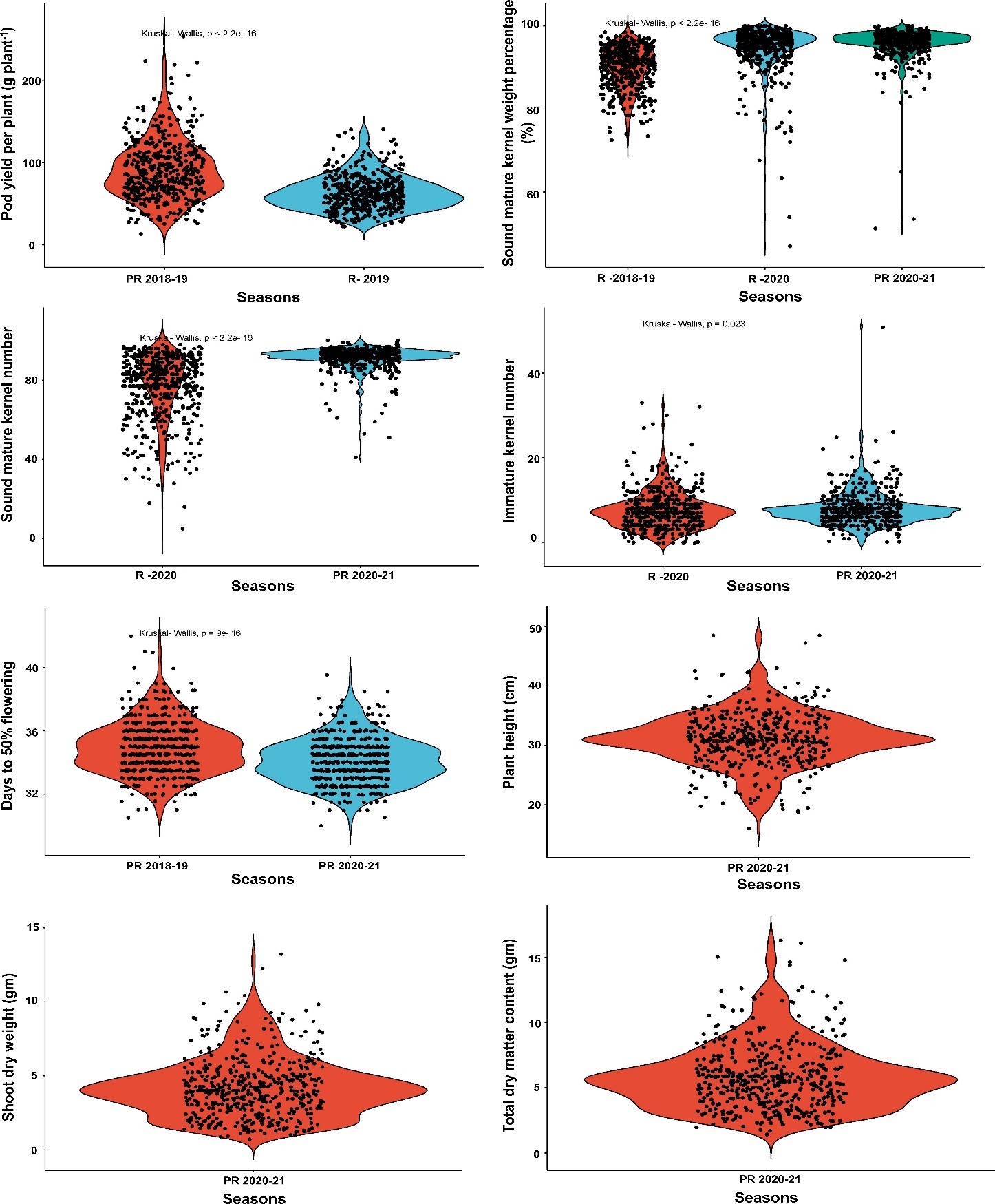


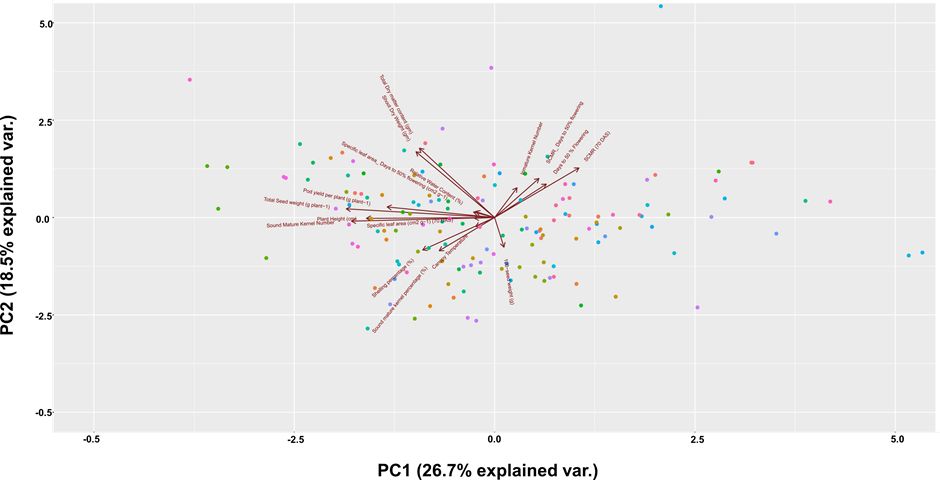


**Figure S3: Principal component analysis of drought tolerance traits:** PCA biplot graph depicts the distribution of genotypes in two main components for drought tolerance traits. The two components accounted 26.7% and 18.5% of the variance respectively. Coloured dots denotes different MLs, and magnitude of the vectors indicates contribution of traits to PC1 and PC2 components

**Figure S4a Distribution of high quality SNPs retained for MAGIC population**


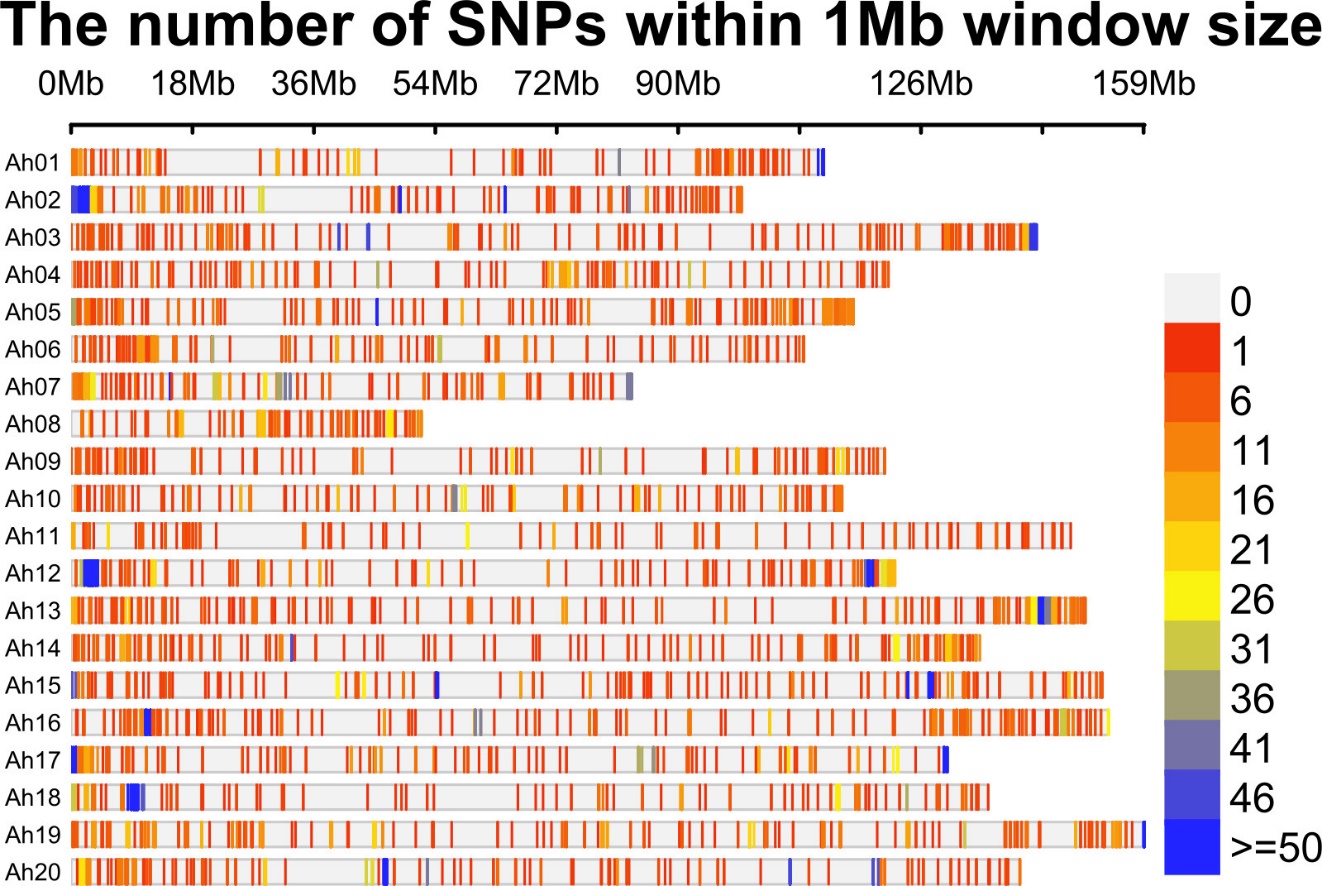


**Figure S4b** **LD decay plot showing the LD decay distance**: An overall LD decay distance of 180 kbp


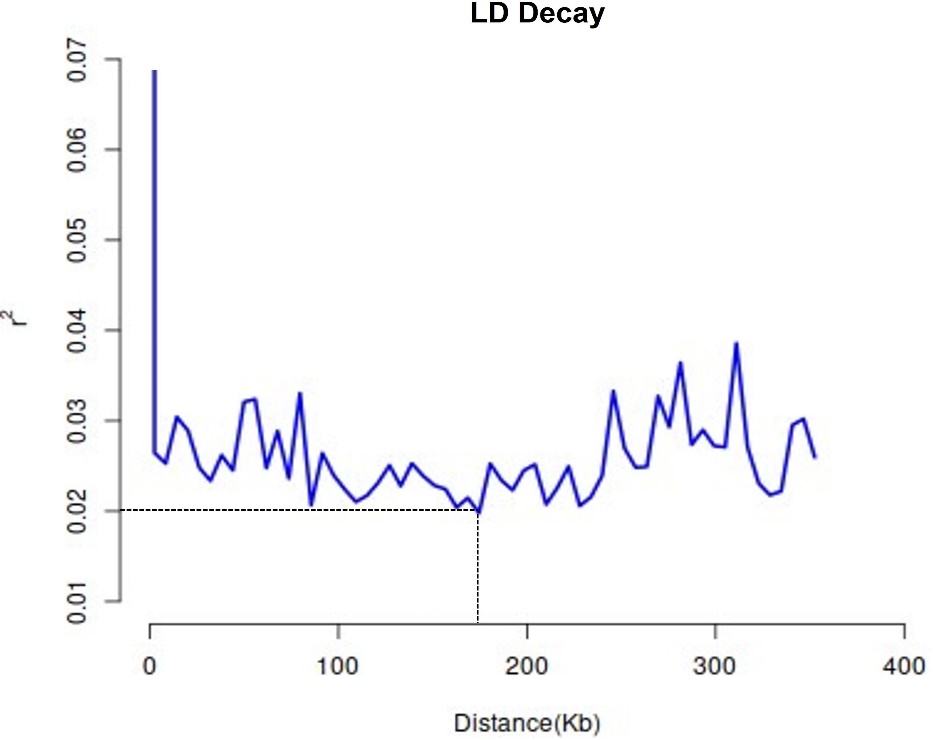


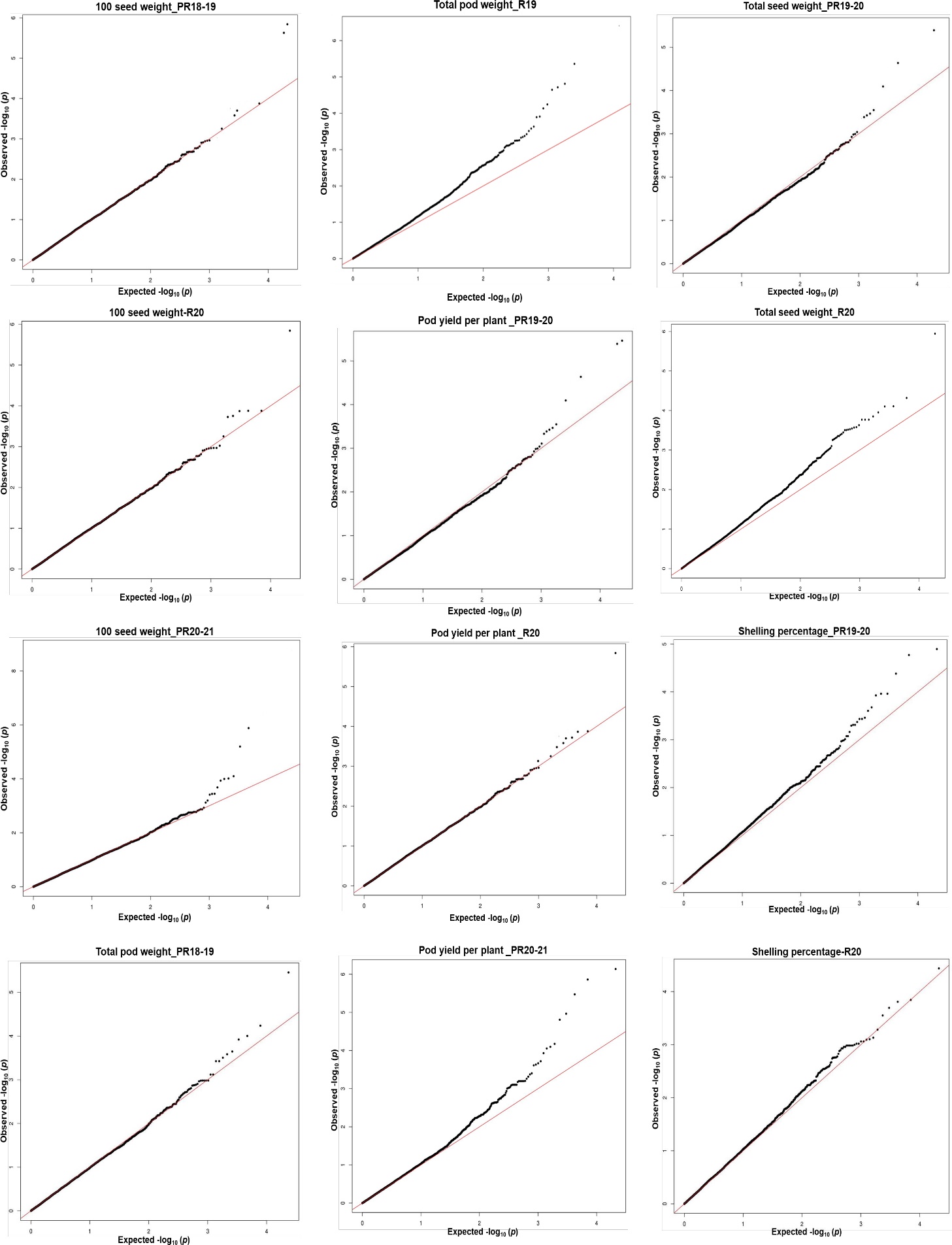


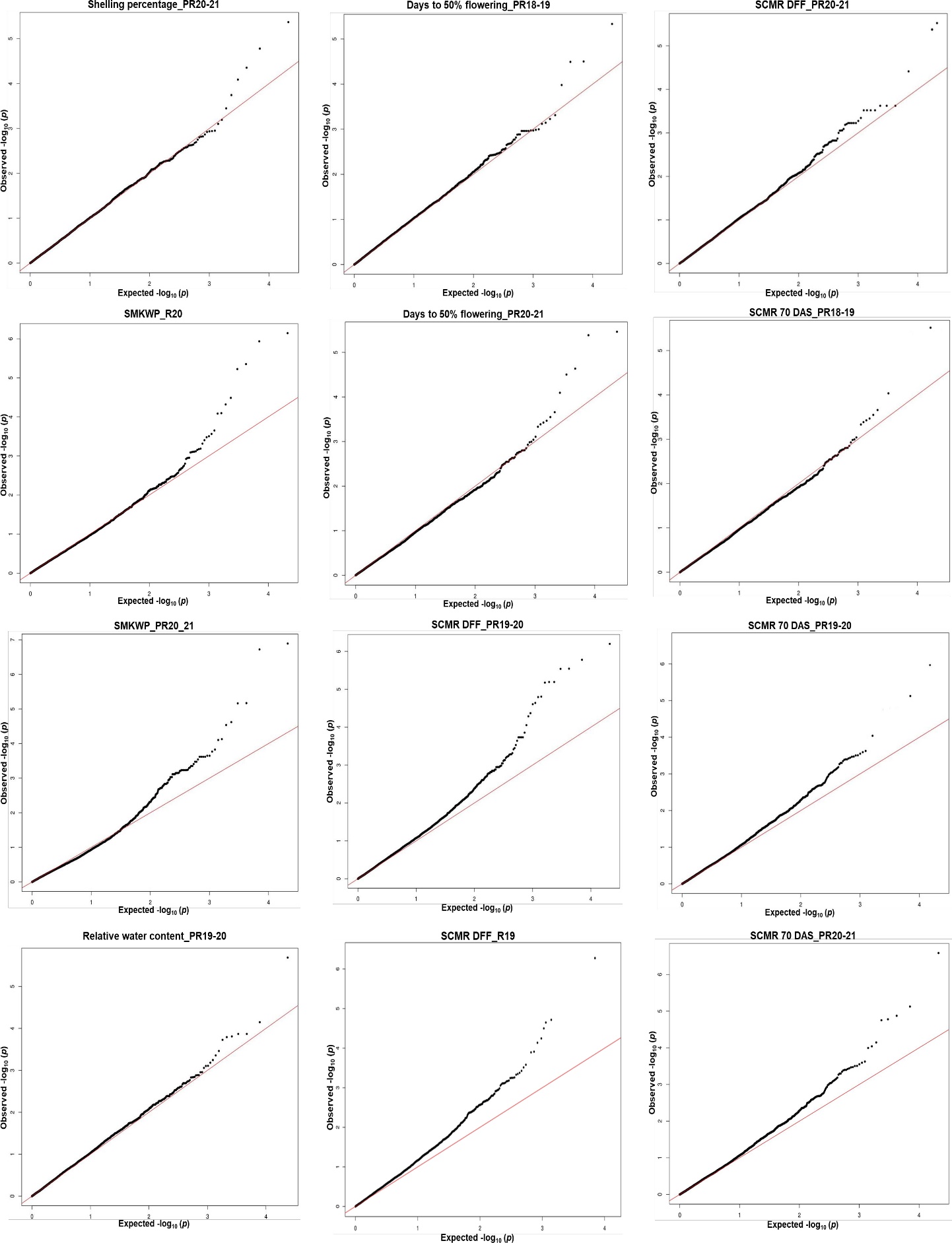


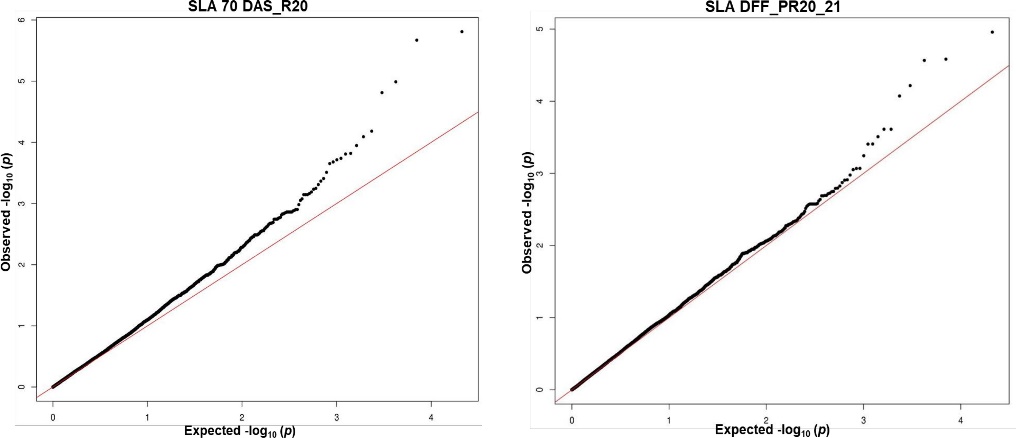


**Figure S5: Q–Q plots of the observed and the expected *p* values of the GWAS model**


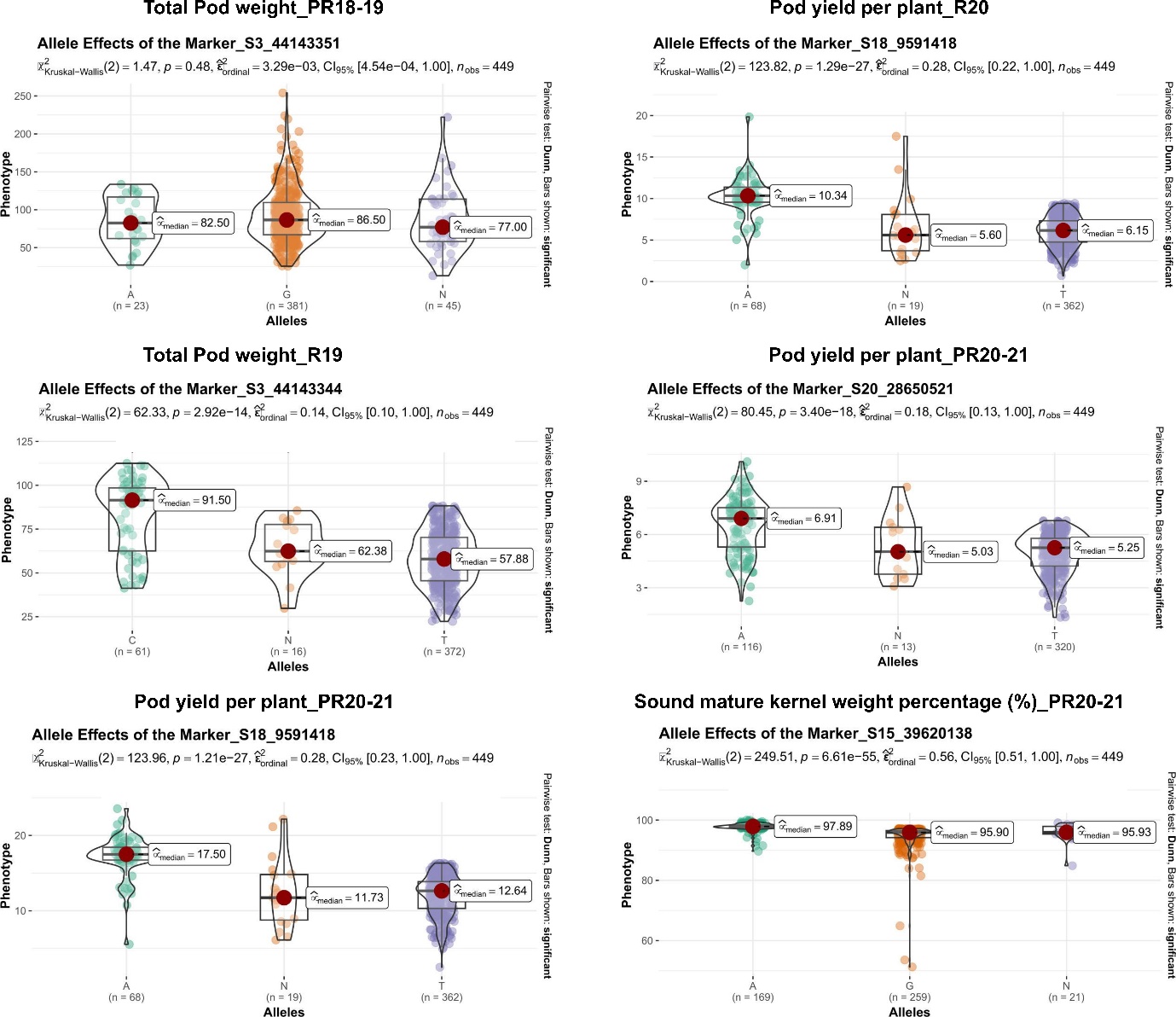


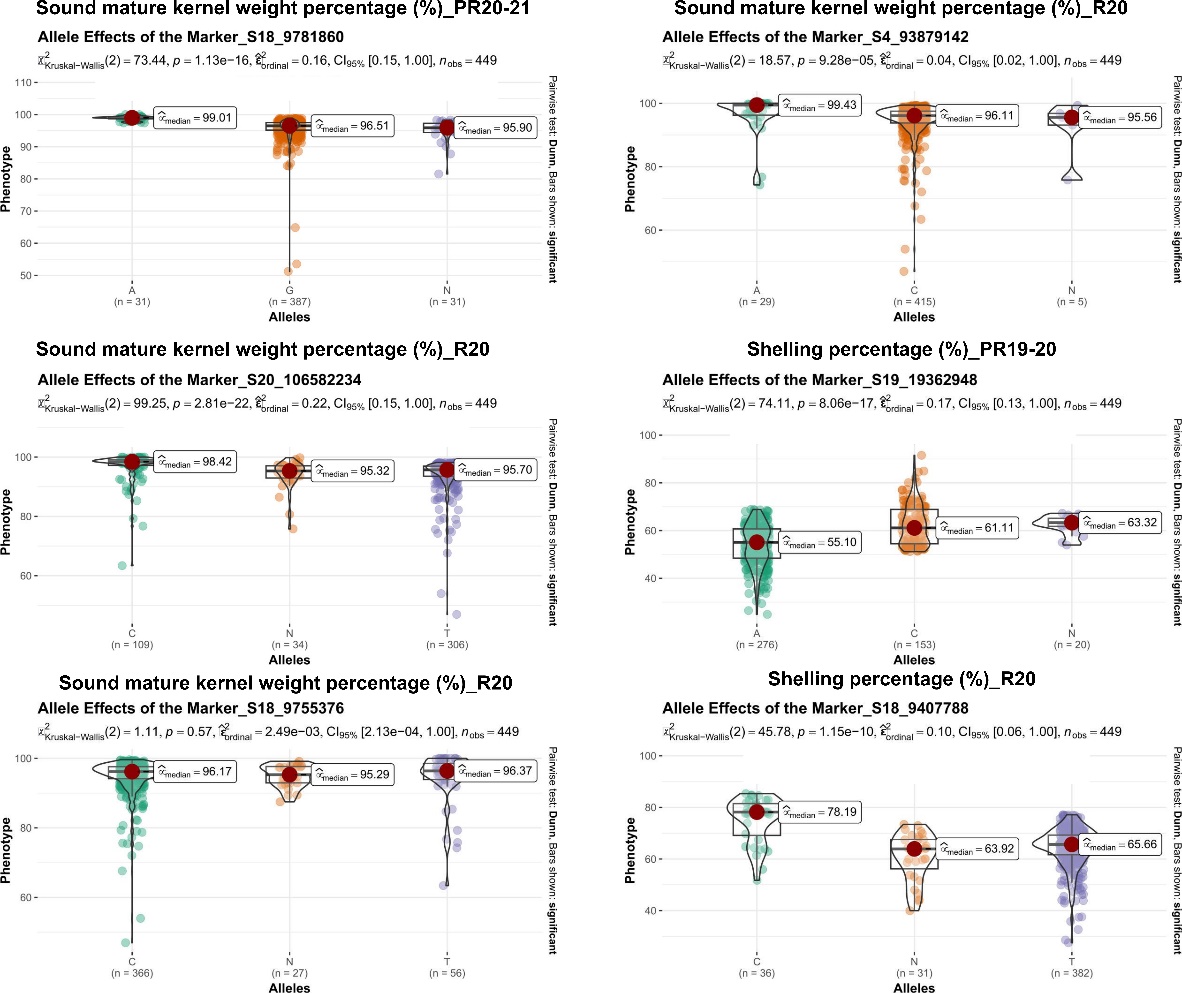


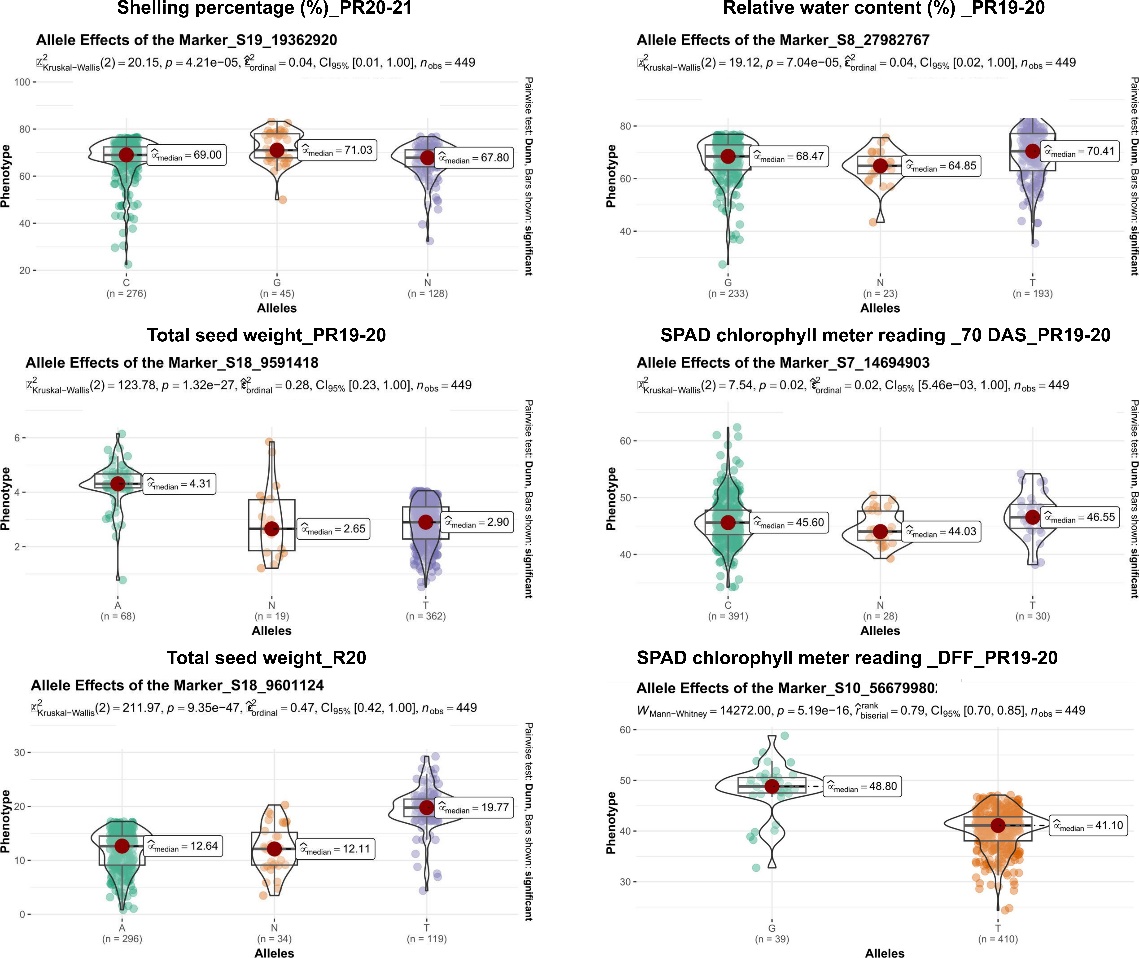


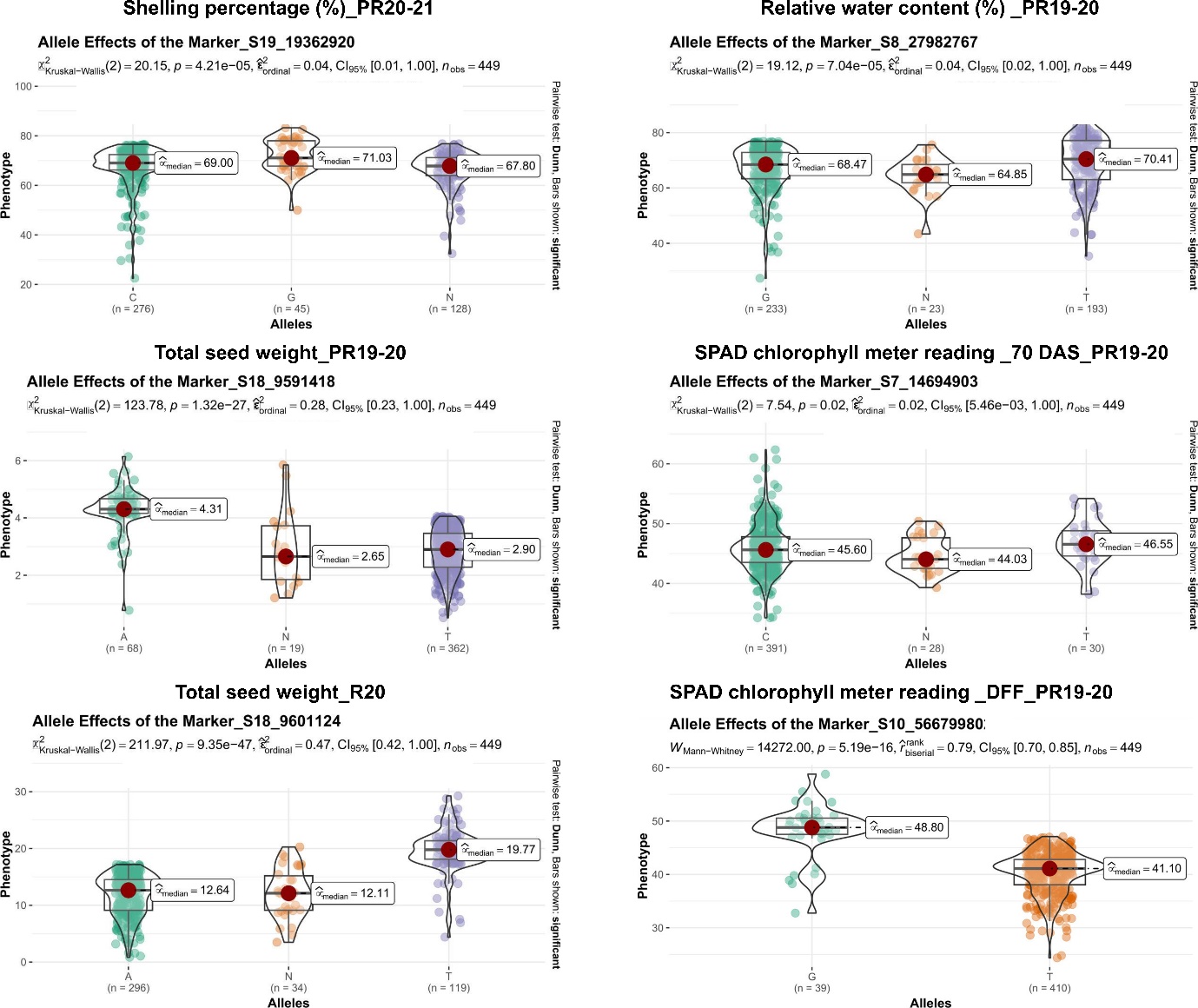


**Figure S6: Phenotypic distribution and allele segregation of associated SNPs:** Phenotypic variations of alleles of associated SNPs for drought tolerance traits
